# Genome-based identification of a putative ulvan-related gene cluster in *Celeribacter* sp.

**DOI:** 10.7717/peerj.21957

**Published:** 2026-11-12

**Authors:** Huynh Hoang Nhu Khanh, Hanh Hong Hoang, Vo Thi Dieu Trang, Hang Thi Thuy Cao

**Affiliations:** 1 Department of Marine Biochemistry and Biotechnology, Institute of Oceanography, Vietnam Academy of Science and Technology, Khanh Hoa, Vietnam; 2 Department of Analytical Chemistry and Technology Development, Institute of Oceanography, Vietnam Academy of Science and Technology, Khanh Hoa, Vietnam

**Keywords:** Ulvan utilization, Polysaccharide utilization locus, Carbohydrate-active enzymes, Marine Alphaproteobacteria, Celeribacter, Sulfatase

## Abstract

Ulvan is a sulfated polysaccharide that constitutes a major component of the cell wall of green macroalgae in the genus *Ulva* and plays an important role in coastal carbon and sulfur cycling. Although ulvan-modifying enzymes and gene systems have been characterized primarily in Bacteroidetes and, to a lesser extent, Gammaproteobacteria, their occurrence in Alphaproteobacteria remains poorly understood. In this study, strain ULVN23_4 was isolated from the surface of *Ulva lactuca* collected in Nha Trang Bay, Vietnam, and its genome was sequenced using the Illumina platform, followed by genome annotation, phylogenomic analysis, and carbohydrate-active enzyme (CAZyme) profiling. Genome-based relatedness analyses using average nucleotide identity (ANI) and digital DNA–DNA hybridization (dDDH) supported its classification as a putative novel genomic species within the genus *Celeribacter*. Genome analysis identified a candidate ulvan-associated gene cluster containing a putative polysaccharide lyase family 25 (PL25)-like protein, a putative glycoside hydrolase family 105 (GH105)-like protein, and an S1-family sulfatase assigned to subfamily 7 (S1_7), co-localized with an adenosine triphosphate-binding cassette transporter. Notably, this locus lacks the canonical starch utilization system proteins C and D (SusC/D) transport pair typically found in Bacteroidetes polysaccharide utilization loci (PULs), suggesting a non-canonical, transporter-associated genomic organization. Plate-based assays produced clear halos on ulvan-containing medium and blue coloration on medium supplemented with 5-bromo-4-chloro-3-indolyl sulfate (X-SO_4_), observations consistent with modification or depolymerization of the ulvan-containing substrate and sulfatase-associated activity, respectively. Collectively, these findings support the hypothesis that this genomic region may participate in the processing of ulvan-derived or related sulfated substrates. This study expands the phylogenetic range of bacteria potentially involved in ulvan transformation and highlights Alphaproteobacteria as an underexplored group in the turnover of sulfated polysaccharides in marine environments. Further biochemical characterization will be required to validate the predicted enzymatic functions.

## Introduction

Polysaccharides from marine macroalgae constitute abundant and renewable carbon reservoirs in coastal ecosystems and have attracted increasing attention for applications in biotechnology, nutraceuticals, and biomedicine (Barahona et al., 2014; Pari et al., 2025). Beyond their applied value, algal polysaccharides are now recognized as key ecological substrates that shape microbial community structure and function, linking microbial metabolism to coastal carbon and sulfur cycling. Recent genome-resolved and ecosystem-scale studies have further suggested that the turnover of complex algal polysaccharides is a major driver of microbial niche differentiation, metabolic specialization, and biogeochemical fluxes in nearshore environments, highlighting the importance of genomic determinants underlying polysaccharide utilization.

Among algal polysaccharides, ulvan is the principal sulfated structural component of green macroalgae belonging to the genus *Ulva* (Ulvaceae), accounting for up to 30% of algal dry weight (Li et al., 2023). Ulvan is a highly heterogeneous polysaccharide primarily composed of repeating disaccharide units of L-rhamnose-3-sulfate (Rha3S) linked to uronic acids (glucuronic or iduronic acid). Xylose is also found in ulvan and may occasionally replace uronic acid residues rather than forming side chains (Tran et al., 2018; Tran et al., 2023). This chemical complexity distinguishes ulvan from other algal sulfated polysaccharides, such as fucoidan, which is dominated by fucose sulfate (Nguyen et al., 2025), or carrageenan, which mainly consists of sulfated galactose residues (Jabeen et al., 2025). In addition to their ecological importance, ulvan and ulvan-derived oligosaccharides display diverse biological activities, including anticoagulant, antioxidant, immunomodulatory, anti-inflammatory, and antitumor effects, reinforcing interest in controlled ulvan depolymerization for functional biomaterials and nutraceutical applications (André et al., 2023; Pari et al., 2025).

Microbial degradation of ulvan requires the coordinated action of multiple enzyme classes, reflecting the sulfate-rich and structurally complex nature of the polymer. Ulvan lyases, particularly those of polysaccharide lyase family PL25, initiate depolymerization by cleaving the Rha3S–uronic acid linkage *via* a β-elimination mechanism, generating unsaturated uronic acid-containing oligosaccharides (Ulaganathan et al., 2017; Reisky et al., 2019). These intermediates are subsequently processed by glycoside hydrolases such as GH105, which remove terminal unsaturated uronic acid residues (Collén et al., 2014). In parallel, sulfatases-especially members of the S1_7 subfamily-play a critical role by removing sulfate esters that otherwise hinder further enzymatic cleavage and downstream carbohydrate metabolism (Stam et al., 2023).

Importantly, recent studies emphasize that efficient ulvan utilization depends not only on the presence of individual enzymes, but also on their genomic colocalization, coordinated regulation, and integration with transport and sensing systems. Genome-resolved analyses have shown that ulvan-modifying enzymes are frequently encoded within discrete genetic loci, often referred to as polysaccharide utilization loci (PULs), which integrate extracellular depolymerization, substrate transport, and intracellular catabolism. To date, well-characterized ulvan-associated PULs have been reported mainly in members of the phylum Bacteroidetes and, to a lesser extent, in certain Gammaproteobacteria, including taxa such as *Formosa agariphila* and *Alteromonas* spp. (Ulaganathan et al., 2017; Ulaganathan et al., 2018; Reisky et al., 2019). These systems have become reference models for understanding ulvan utilization at the genomic and biochemical levels. However, the PUL concept has been largely established based on Bacteroidetes, and comparative genomics increasingly suggests that polysaccharide utilization strategies can vary substantially across bacterial lineages, including the emergence of non-canonical, PUL-like genomic architectures outside classical frameworks.

Despite the recognized ecological and biotechnological importance of ulvan, knowledge of its microbial transformation remains strongly biased toward a limited number of bacterial groups. In particular, the ulvan-degrading potential of Alphaproteobacteria remains poorly documented. This gap is notable given that Alphaproteobacteria - especially members of the Roseobacteraceae are abundant, metabolically versatile components of marine microbial communities and are frequently associated with macroalgae and phytoplankton-derived organic matter. Whether these bacteria harbor ulvan-associated enzyme systems, and how such systems are organized and regulated at the genome level, remains largely unresolved.

Recent advances in genome sequencing, comparative genomics, and carbohydrate-active enzyme (CAZyme) annotation have greatly expanded the capacity to identify previously unrecognized polysaccharide utilization systems and to explore PUL-like architectures beyond established model taxa (Koch et al., 2019; Chen et al., 2024). Genome-based approaches are particularly valuable for uncovering candidate polysaccharide degradation loci in bacterial lineages for which biochemical characterization is still limited, and for placing polysaccharide metabolism within an evolutionary and genomic context.

In this study, strain ULVN23_4 was isolated from the surface of *Ulva lactuca* collected in Nha Trang Bay, Vietnam, and its draft genome was sequenced and comparatively analyzed. The aim of this study was to investigate the genomic basis of ulvan utilization in this strain, with a particular focus on identifying putative polysaccharide utilization loci (PUL)-like systems and assessing their organization within a phylogenomic context. This work also seeks to explore the diversity of ulvan-associated loci beyond the classical Bacteroidetes model.

## Materials & Methods

### Strain isolation and enzymatic screening

Surface thalli of *Ulva lactuca* were collected from an accessible coastal intertidal site in Nha Trang, Vietnam (12°15′22.5″N, 109°11′52.3″E), in March 2024. The collection involved a small quantity of a common, non-protected macroalgal species and did not involve entry into a restricted-access area. Sampling was conducted as part of the institutionally approved research project “Research on the Discovery of bacterial genes encoding marine polysaccharide converting enzymes and recombinant expression for the development of biomedical products (grant number: VAST02.01/24-25)” under Decision No. 1264/QÐ-VHL, issued by Vietnam Academy of Science and Technology (VAST) on 26th June, 2023. No separate field-collection permit was required or issued for this activity.

Samples were transported on ice and processed within 12 h. The thalli were rinsed thoroughly with sterile seawater to remove loosely attached epiphytes and debris. Approximately 10 g of wet biomass was incubated in 100 mL of sterile seawater at 30 °C for 10 days. Aliquots (100 µL) were plated onto agar medium containing sea salts (28 g/L) and 1% (w/v) ulvan (MBU medium, pH 7.4–7.8), and incubated at 30 °C for 5 days. Ulvan was extracted from *U. lactuca* using hot-water extraction followed by ethanol precipitation (Hang et al., 2023), with minor modifications. Colonies producing clear halos on cetylpyridinium chloride (cetavlon)-treated ulvan plates were selected as potential ulvan degraders (Silchenko et al., 2015). For this assay, strain ULVN23_4 (PS) was tested alongside a bacterial strain without detectable activity on ulvan-containing agar (NS), which served as a negative comparator under the same assay conditions. Clear zones following cetavlon treatment were interpreted as evidence consistent with modification or depolymerization of the ulvan-containing substrate.

Cell extracts were prepared in 20 mM Tris–HCl buffer containing 125 mM NaCl (pH 6.5). Sulfatase activity was screened on X-SO_4_-containing agar following Mikkelsen et al. (2021). Commercial sulfatase (Sigma) and extraction buffer alone served as positive and negative controls, respectively. A cell extract from a bacterial strain without detectable sulfatase activity was included as an additional negative control. Blue precipitate formation indicated hydrolysis of X-SO_4_. Each assay was independently repeated at least three times, and representative results are shown in Fig. 1. Because the assays were designed as preliminary qualitative screening tests, halo diameters and quantitative statistical analyses were not performed.

**Figure 1:**
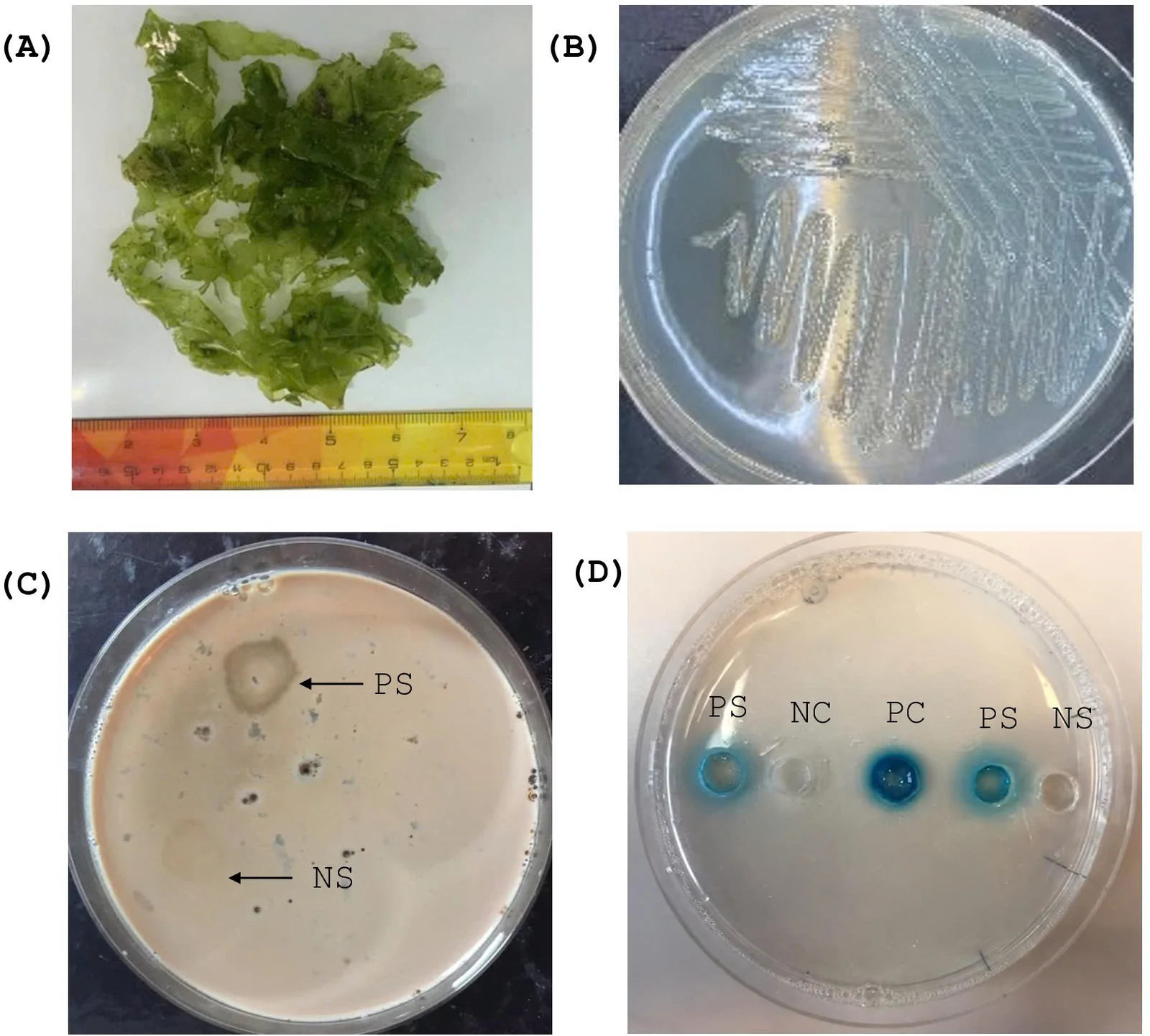
Isolation and qualitative screening of *Celeribacter* sp. ULVN23_4 for ulvan-modifying and sulfatase activities. (A) Thalli of *Ulva lactuca* collected from Nha Trang Bay, Vietnam, used for bacterial isolation and ulvan extraction. (B) Growth of strain ULVN23_4 on marine agar. (C) Screening on ulvan-containing agar following cetavlon treatment. PS indicates the position of strain ULVN23_4, where a clear zone is consistent with modification or depolymerization of the ulvan-containing substrate; NS indicates the position of a bacterial strain with no detectable activity in this assay. Arrows identify the corresponding assay sites (D) Screening of cell extracts for sulfatase activity using 5-bromo-4-chloro-3-indolyl sulfate (X-SO_4_). PS, cell extract of strain ULVN23_4 tested in two technical replicate wells; NS, cell extract of a bacterial strain with no detectable sulfatase activity; PC, positive control containing commercial sulfatase from Sigma; NC, negative control containing extraction buffer alone. Blue coloration indicates hydrolysis of the chromogenic sulfate ester substrate.

### Genome annotation and phylogenomic analyses

Genomic DNA of strain ULVN23_4 was extracted using the NucleoSpin DNA extraction kit (Clontech, USA). DNA quality was checked by agarose gel electrophoresis, and concentration was determined with a NanoDrop spectrophotometer (Thermo Fisher Scientific, USA). Whole-genome sequencing was carried out on the Illumina HiSeq platform (Illumina, USA). The average sequencing coverage was approximately 61 ×. Annotation was performed using the RAST server (Aziz et al., 2008), Bakta v1.12.0 (database version 6.0) and DFAST (Tanizawa, Fujisawa & Nakamura, 2018), the Bacterial and Viral Bioinformatics Resource Center (BV-BRC; https://www.bv-brc.org/). Functional annotation was conducted with eggNOG-mapper v6.0 (Hernández-Plaza et al., 2023) for Gene Ontology (GO) terms and Cluster of Orthologous Groups (COG) classification.

Phylogenetic analyses were conducted based on both the 16S rRNA gene sequence and genome-wide core genes. The 16S rRNA gene sequence of strain ULVN23_4 was compared with those in the NCBI database using BLASTn to identify closely related taxa. Representative 16S rRNA gene sequences of closely related species were retrieved and aligned using ClustalW implemented in MEGA version 11 (Tamura, Stecher & Kumar, 2021). A phylogenetic tree was constructed using the Neighbor-Joining (NJ) method, and the robustness of the tree topology was evaluated using bootstrap analysis with 1,000 replicates.

For higher-resolution phylogenomic analysis, the Up-to-date Bacterial Core Gene (UBCG) pipeline (Na et al., 2018) was employed using the genome of strain ULVN23_4 together with 53 reference genomes retrieved from the Genome Taxonomy Database (GTDB). A total of 92 conserved bacterial core genes were extracted and individually aligned using MAFFT v7, followed by concatenation into a single alignment. A maximum-likelihood phylogenomic tree was generated using the RAxML algorithm implemented in UBCG with the GTRGAMMA model and 1000 bootstrap replicates. The resulting tree was visualized using the Interactive Tree of Life (iTOL) (https://itol.embl.de/).

Genome-wide relatedness was evaluated using the OrthoANI algorithm implemented in the OAT software (Orthologous ANI Tool) (Lee et al., 2016) and the Genome-to-Genome Distance Calculator (GGDC v3.0; http://ggdc.dsmz.de) (Meier-Kolthoff et al., 2013). ANI values were visualized as a table, following the genome order in the UBCG tree. For dDDH, GGDC Formula 2 (GBDP formula d4) was applied, with a threshold of 70% for species delineation (Wayne et al., 1987).

Signal peptide prediction was not performed in the present study.

### Prediction of carbohydrate-active enzymes and sulfatase

Initial carbohydrate-active enzymes (CAZymes) annotation was performed using the dbCAN3 meta server (accessed 9 July 2025). HMMER searches were conducted against the dbCAN HMM database (dbCAN HMMdb v13) using the recommended thresholds of *E*-value < 1 × 10^−15^ and HMM coverage > 0.35. DIAMOND searches were performed against the CAZy database using the default dbCAN3 threshold (*E*-value < 1 × 10^−102^), and dbCAN-sub was used for CAZyme subfamily annotation with the recommended HMMER thresholds. Candidate annotations supported by at least one dbCAN3 method were manually inspected for domain coverage, protein length, consistency among dbCAN3 outputs, and agreement with conserved-domain annotations before inclusion in the final CAZyme inventory. Under this standard annotation workflow, no polysaccharide lyase (PL) family was assigned to LOCUS_20870. Because no confident PL family was detected using the recommended dbCAN3 thresholds, LOCUS_20870 was selected for further investigation following an exploratory BLASTp similarity search against a curated set of experimentally characterized PL25 ulvan lyases. The candidate was subsequently evaluated using conserved-domain annotation, multiple-sequence alignment, phylogenetic analysis, and structural comparison. The BLASTp search served only as an exploratory similarity search to identify a candidate for further investigation and was not used as evidence for CAZyme family assignment.

Proteins annotated as sulfatases in RAST were further classified using the SulfAtlas database (Barbeyron et al., 2016). Searches were performed with HMMER v3 against SulfAtlas HMM profiles, and the best-scoring hit was used to assign families (S1–S4). For S1 proteins, subfamily classification (S1_n) followed SulfAtlas criteria.

### Identification of polysaccharide utilization loci

Candidate polysaccharide utilization loci (PULs) were predicted using the dbCAN-PUL server. Gene clusters were required to include at least one carbohydrate-active enzyme (CAZyme) and an ATP-binding cassette (ABC) transporter, classified within the Transporter Classification (TC) system. The presence of transcriptional regulators was considered supportive when detected. Predictions were cross-checked with the PULDB database (Stam et al., 2023). Gene cluster maps were visualized using the Chiplot server (https://www.chiplot.online/).

The 17 genomes represent all publicly available *Celeribacter* assemblies included in the genome phylogeny and encompass the closest relatives of strain ULVN23_4. The amino acid sequences of LOCUS_20870, LOCUS_20900, and LOCUS_20920 were used as BLASTp queries. Homologs were retained using an *E*-value threshold of 1 × 10^−10^, a minimum query coverage of 60%, and a minimum amino acid identity of 25%. For each retained homolog, 10 upstream and 10 downstream protein-coding genes were retrieved from the corresponding GenBank annotation. The resulting genomic regions were compared for gene content, transcriptional orientation, and the co-localization of carbohydrate-active enzymes, sulfatases, and transporter components. Representative regions were visualized using clinker (version 0.0.32) (https://github.com/gamcil/clinker/blob/master/README.md).

### Protein sequence and structural analyses

Genes LOCUS_20870, LOCUS_20900, and LOCUS_20920, predicted to encode proteins potentially involved in the processing of ulvan-derived substrates, were selected for sequence and structural analysis. Multiple sequence alignments (MSAs) were generated using Clustal Omega (Sievers et al., 2011) and MAFFT v7. Alignments were visualized with ESPript 3.0 (https://espript.ibcp.fr/ESPript/ESPript/). Phylogenetic trees were reconstructed with MEGA 11 (Tamura, Stecher & Kumar, 2021) using the maximum likelihood method and 1000 bootstrap replicates. Pairwise sequence identities were calculated.

AlphaFold2 modeling was explored for the candidate proteins. Because the model generated for LOCUS_20870 was insufficiently reliable for functional interpretation, it was not used as structural evidence. Detailed structural analysis was retained for the S1_7 sulfatase candidate LOCUS_20920 and was visualized using PyMOL v2.5. Structural homology modeling was additionally conducted using SWISS-MODEL. Functional domains were annotated with Pfam and InterProScan (Jones et al., 2014).

## Results

### Screening of seaweed polysaccharide-degrading activity

Strain ULVN23_4 produced clear halos on ulvan-containing agar plates following cetavlon treatment and blue pigmentation on X-SO_4_-supplemented medium (Fig. 1). The formation of clear halos was consistent with modification or depolymerization of the ulvan-containing substrate under the assay conditions, whereas the blue coloration indicated sulfatase-associated activity toward the chromogenic sulfate ester substrate. These qualitative plate assays provide preliminary strain-level phenotypic evidence but do not establish ulvan-specific enzyme activity or link the observed phenotypes to the candidate genes identified in the genome. Therefore, the biochemical basis, substrate specificity, and contribution of the predicted gene cluster remain to be determined through targeted enzymatic and molecular validation.

### General genome features

The draft genome of *Celeribacter* sp. ULVN23_4 is 3,987,018 bp in size, assembled into 37 contigs, with an N50 of 387,787 bp and an L50 of 3 (Table 1). The draft genome assembly was obtained with an estimated average sequencing coverage of approximately 61x. The genome has a GC content of 59.2%, with 100% completeness and 0% contamination as assessed by CheckM, indicating a high-quality assembly (Parks et al., 2015). A total of 3,840 coding sequences (CDSs) were predicted, along with 46 tRNA genes, three rRNA genes, one transfer-messenger RNA (tmRNA), and 18 non-coding RNAs (ncRNAs). No CRISPR arrays were detected. The coding density was 89.2%, consistent with other members of the genus *Celeribacter*. Potential plasmid sequences were screened using PlasmidFinder, and no plasmid-associated contigs were detected in the ULVN23_4 genome.

**Table 1:** General genome statistics of *Celeribacter* sp. ULVN23_4. Values are based on the draft genome assembly of strain ULVN23_4.

**Feature**	***Celeribacter*** ** sp. ULVN23_4**
Genome Size (bp)	3,987,018
GC content (%)	59.2
Contig count	37
Completeness (%)	100
Contamination (%)	0
Contigs N50 (bp)	387,787
Contigs L50	3
Gap Ratio (%)	0.0
CDS	3,840
tRNA	46
rRNA	3
CRISPRs	0
Coding density (%)	89.2

### Phylogenomic positioning and taxonomic assignment

To determine the taxonomic position of strain ULVN23_4, phylogenetic analyses were conducted based on both the 16S rRNA gene sequence and whole-genome data. The 16S rRNA gene sequence was compared using BLASTn, and closely related reference sequences were selected to construct a phylogenetic tree (Fig. 2A). The resulting tree placed strain ULVN23_4 within the genus *Celeribacter*, forming a cluster with other members of this genus. Notably, ULVN23_4 showed the closest relationship to *Celeribacter neptunius* H14, although the bootstrap support for this node was relatively low (42%), indicating limited resolution at the species level.

**Figure 2:**
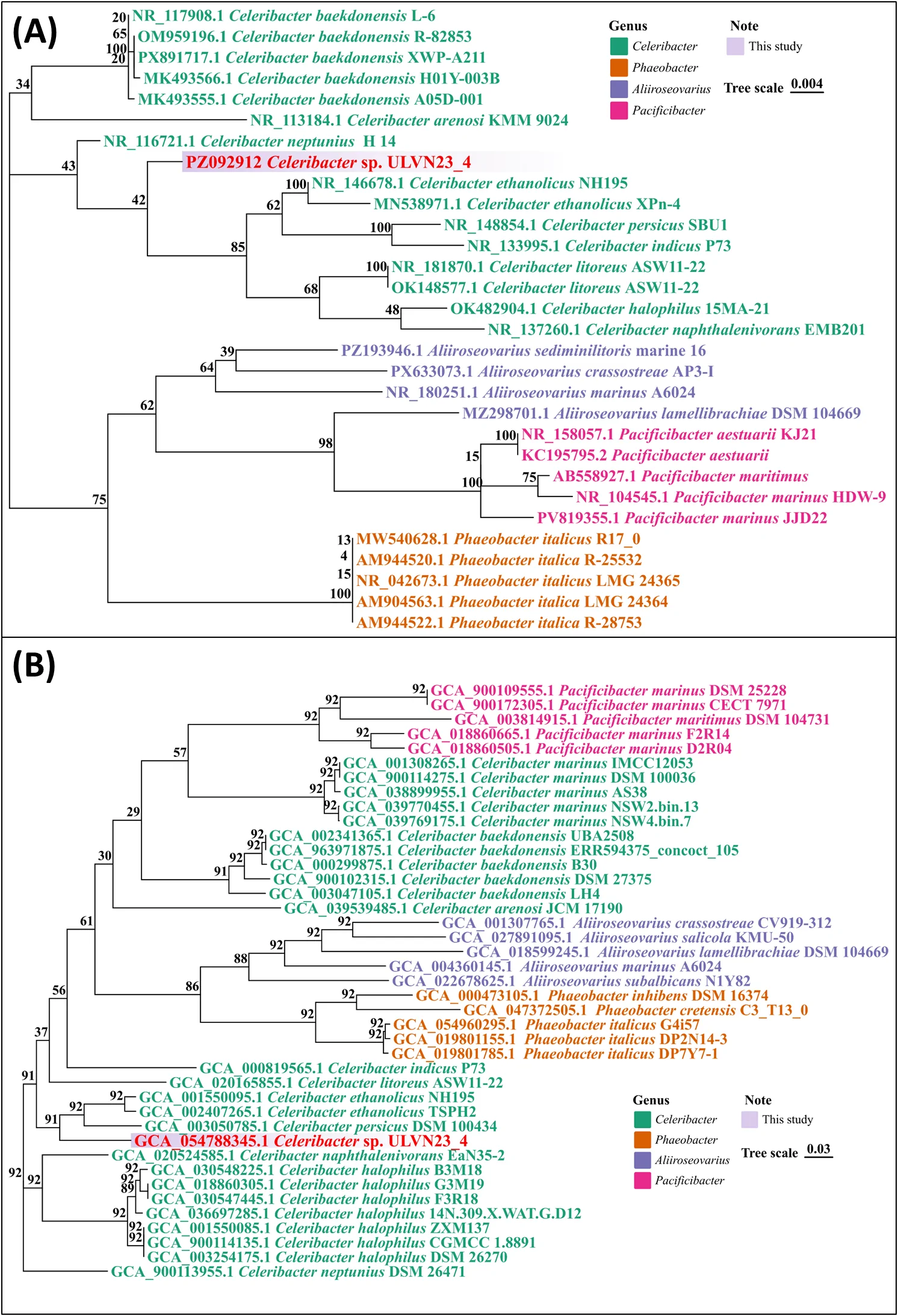
Phylogenetic position and genomic relationships of *Celeribacter* sp. ULVN23_4. (A) Phylogenetic tree based on the 16S rRNA gene sequence. (B) Maximum-likelihood phylogenomic tree reconstructed from 92 core genes using the UBCG pipeline. ULVN23_4 (highlighted in red) clusters within the genus *Celeribacter*. Pairwise average nucleotide identity (ANI) and digital DNA–DNA hybridization (dDDH; formula d4) values summarized in Table 2 were used to assess genomic relatedness among the analyzed strains.

To further resolve its phylogenetic position, a genome-based phylogenetic analysis was performed using the UBCG pipeline with 54 *Celeribacter* genomes, including strain ULVN23_4 (Fig. 2B). The resulting tree showed that ULVN23_4 is most closely related to *Celeribacter persicus* DSM 100434, with a relatively high bootstrap value (91%), suggesting a more robust phylogenetic relationship. In addition, genome-based taxonomic classification using GTDB-Tk assigned strain ULVN23_4 to the genus *Celeribacter* within the class Alphaproteobacteria.

Pairwise whole-genome similarity analyses further supported its distinct taxonomic status. The OrthoANI values between ULVN23_4 and closely related *Celeribacter* strains ranged from 76.76% (with *C. indicus* P73) to 82.67% (with *C. ethanolicus* NH195), while the corresponding dDDH values ranged from 19.1% to 42.5% (Table 2). These values are well below the accepted thresholds for species delineation (ANI ≥ 95% and dDDH ≥ 70% (Chun et al., 2018), indicating that strain ULVN23_4 likely represents a novel genomic species within the genus *Celeribacter*.

**Table 2:** Genomic relatedness of *Celeribacter* sp. ULVN23_4 to closely related species based on OrthoANI and digital DNA–DNA hybridization (dDDH). Pairwise average nucleotide identity (ANI) and digital DNA–DNA hybridization (dDDH) values were calculated using OrthoANI and the Genome-to-Genome Distance Calculator (GGDC), respectively. Species delineation thresholds are generally considered as ANI ≥ 95% and dDDH ≥ 70%.

**Genomes**	**OrthoANI**	**dDDH**
*Celeribacter halophilus ZXM137*	78.63	25.2
*Celeribacter ethanolicus NH195*	82.67	42.5
*Celeribacter ethanolicus TSPH2*	82.46	41
*Celeribacter persicus DSM 100434*	82.18	38.7
*Celeribacter halophilus DSM 26270*	78.74	25.4
*Celeribacter halophilus G3M19*	78.63	26
*Celeribacter litoreus ASW11-22*	77.21	23.9
*Celeribacter naphthalenivorans EaN35-2*	80.77	33.8
*Celeribacter halophilus F3R18*	78.52	26
*Celeribacter halophilus B3M18*	78.53	25.7
*Celeribacter halophilus 14N.309.X.WAT.G.D12*	78.77	26
*Celeribacter indicus* P73	76.76	19.1
*Celeribacter neptunius DSM 26471*	79.33	27.5
*Celeribacter halophilus CGMCC 1.8891*	78.71	25.4

### Functional genome annotation of *Celeribacter* sp. ULVN23_4

Genome annotation indicated that putative functions were assigned to 61.4% of the predicted protein-coding genes based on homology searches against multiple databases. COG classification (Fig. 3A) showed that the most abundant categories were amino acid transport and metabolism (E), inorganic ion transport and metabolism (P), transcription (K), and energy production and conversion (C), each represented by more than 200 genes. The abundance of category C genes reflects a substantial repertoire associated with energy production and conversion but does not by itself indicate a specific capacity for polysaccharide degradation. Similar distributions have been reported for other *Celeribacter* genomes, including *C. indicus* P73, *C. marinus* IMCC12053, and *C. neptunius* DSM 26471, reflecting ecological adaptation of this genus to diverse marine niches (Wang et al., 2020; Liang et al., 2024).

**Figure 3:**
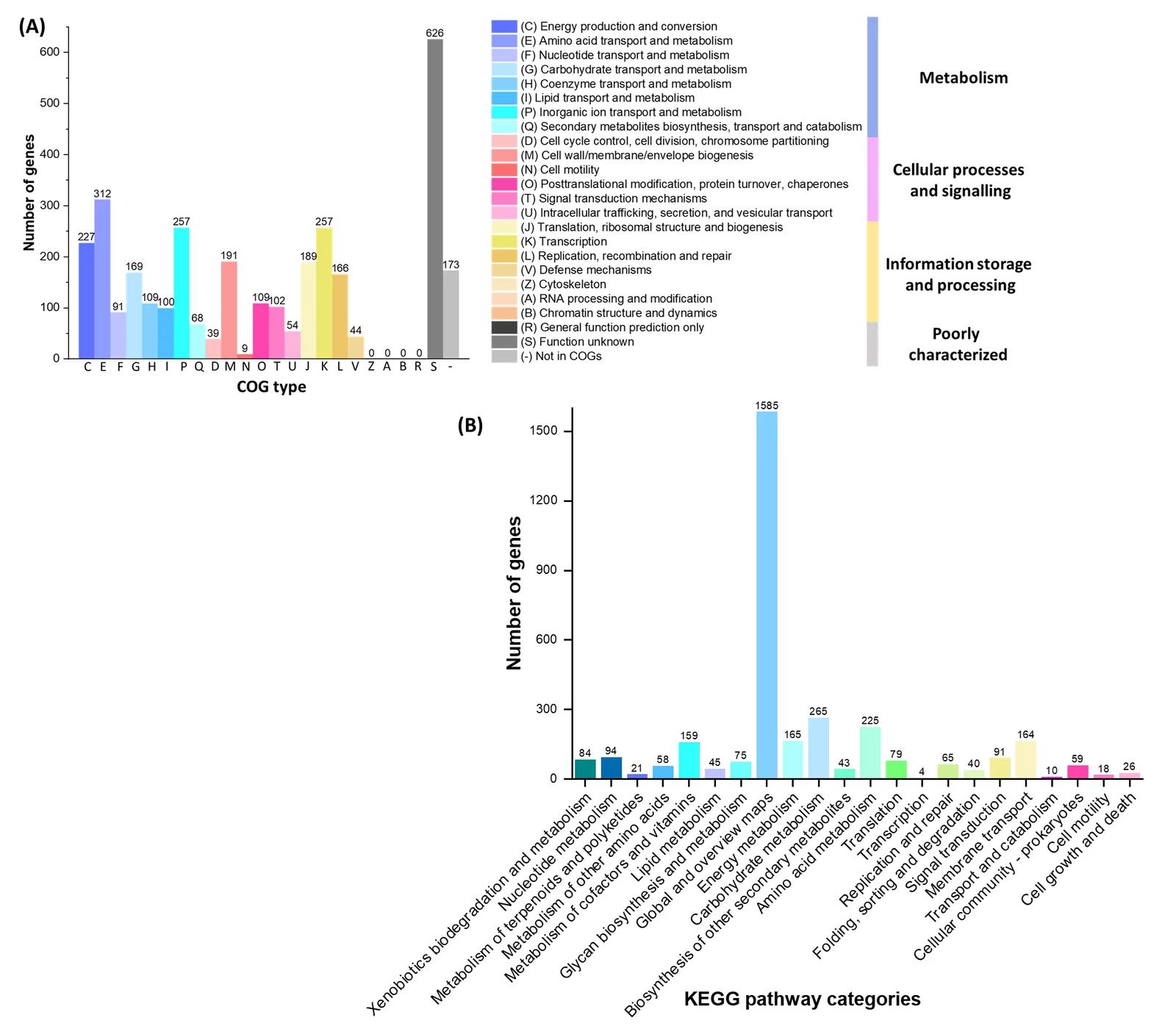
Functional annotation of the *Celeribacter* sp. ULVN23_4 genome. (A) Distribution of predicted genes among COG functional categories. Category letters are shown on the *x*-axis, with their corresponding functional descriptions and broader functional classes displayed to the right of panel A. Both legend elements apply exclusively to (A). (B) Distribution of predicted genes among KEGG functional categories, with each category identified directly on the *x*-axis. Colors distinguish individual bars and do not indicate additional functional groupings.

Genes in the carbohydrate transport and metabolism category (G) accounted for 169 genes (5.13%), a proportion comparable to *Celeribacter* sp. PS-C1 (5.86%), a strain reported to degrade lignocellulosic biomass (Radzlin et al., 2022). This enrichment in category G suggests the presence of transporters, hydrolases, and kinases that may collectively support carbohydrate utilization, consistent with the observed capacity of ULVN23_4 to act on marine polysaccharides. At the broader functional level, the genome profile was in line with other *Celeribacter* genomes, with genes distributed across metabolism (30.3–46.6%), cellular processes and signaling (12.2–18.3%), information storage and processing (12.4–20.1%), and poorly characterized functions (15.2–19.0%) (Radzlin et al., 2022). ULVN23_4 had a relatively high proportion of genes assigned to the “function unknown” category (19.0%), suggesting that a substantial fraction of its coding potential remains uncharacterized.

Complementary KEGG annotation (Fig. 3B) supported that most genes were involved in carbohydrate, amino acid, and energy metabolism. KEGG annotation showed that many genes were assigned to carbohydrate, amino-acid, and energy metabolism, as well as membrane transport. These broad functional categories indicate general metabolic versatility but do not establish a dedicated ulvan-utilization pathway. A particularly high proportion of carbohydrate metabolism genes further supports the versatile use of diverse sugars, including complex polysaccharides common in marine environments. However, no dedicated pathways for sulfated polysaccharides (*e.g.*, fucoidan, carrageenan, ulvan) were detected. This limitation may reflect (i) the incomplete representation of rare marine enzymes in KEGG (Alma’abadi, Gojobori & Mineta, 2015), or (ii) the presence of genes that remain functionally uncharacterized and are classified under “function unknown” (Ellis, 2017). However, this result should be interpreted cautiously because KEGG currently does not contain a dedicated pathway entry for ulvan metabolism. Degradation of ulvan is typically inferred from the presence of carbohydrate-active enzymes such as polysaccharide lyases, glycoside hydrolases, and sulfatases rather than from KEGG pathway annotations. Therefore, the absence of a KEGG-annotated ulvan pathway does not necessarily indicate the absence of ulvan degradation capability. Consequently, KEGG annotation alone is insufficient to establish ulvan-degrading potential, emphasizing the need for complementary approaches such as CAZyme profiling (‘Identification of carbohydrate-active enzymes (CAZymes) and sulfatase’), PUL prediction (‘Prediction of polysaccharide utilization loci (PUL)’), and biochemical validation.

### Identification of carbohydrate-active enzymes (CAZymes) and sulfatase

The dbCAN server was used to annotate genes encoding carbohydrate-active enzymes (CAZymes) in the genome of *Celeribacter* sp. ULVN23_4. In total, 151 CAZymes were identified, distributed among glycoside hydrolases (GH, 34.2%), glycosyltransferases (GT, 45.2%), auxiliary activity enzymes (AA, 5.2%), carbohydrate-binding modules (CBM, 11.0%), and carbohydrate esterases (CE, 4.5%) (Fig. 4). As in other *Celeribacter* genomes, no polysaccharide lyases were detected through automated annotation (Radzlin et al., 2022).

**Figure 4:**
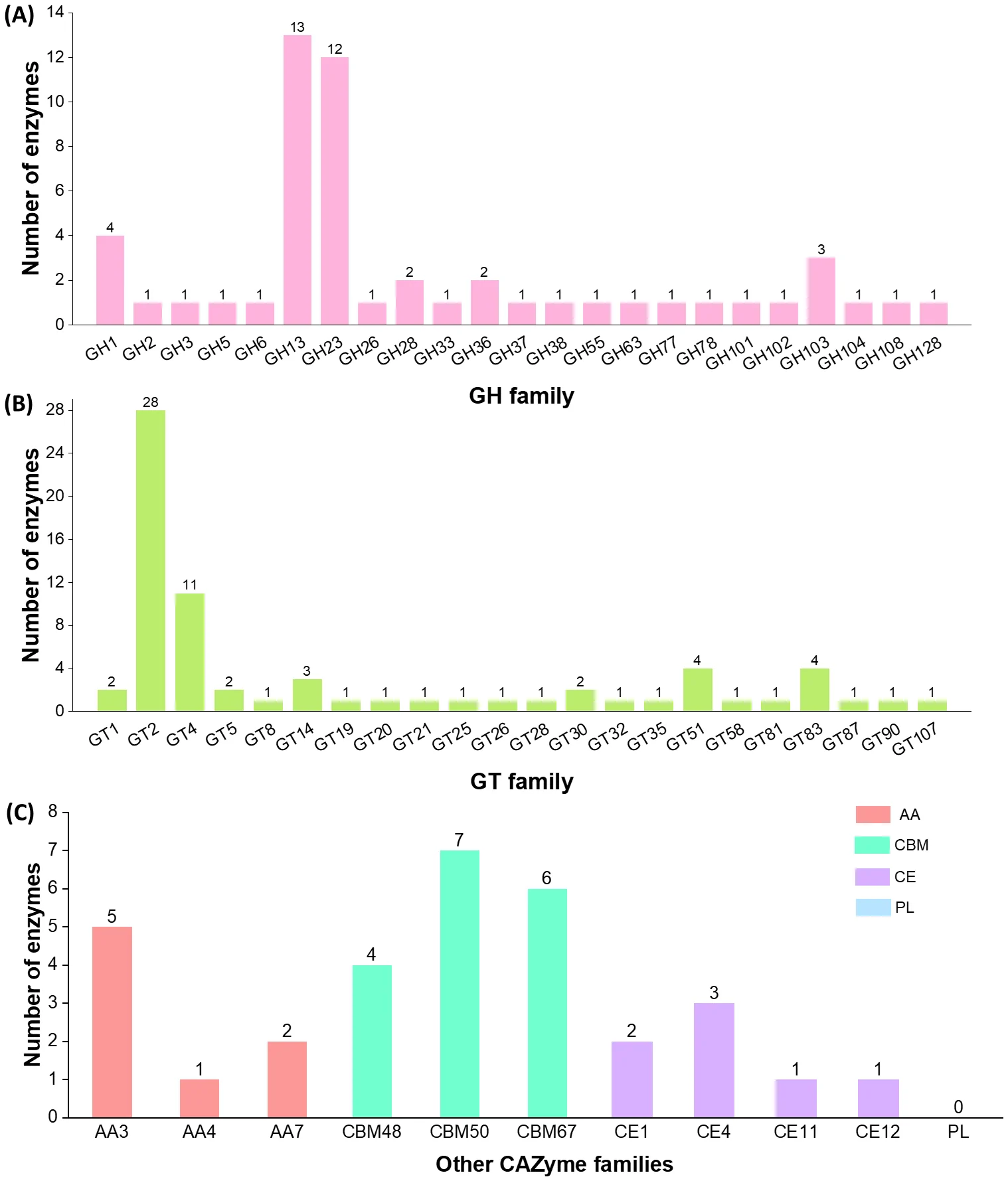
Distribution of predicted carbohydrate-active enzymes (CAZymes) in the *Celeribacter* sp. ULVN23_4 genome. (A) Distribution of glycoside hydrolase (GH) families. (B) Distribution of glycosyltransferase (GT) families. (C) Distribution of other CAZyme families, including auxiliary activities (AA), carbohydrate-binding modules (CBM), and carbohydrate esterases (CE). The *x*-axis labels identify the CAZyme families, except for PL in (C), which represents the polysaccharide lyase class. Bar heights and numerical annotations indicate the number of predicted enzymes assigned to each family or class. Colors in (C) distinguish AA, CBM, CE, and PL. The PL entry is shown with a count of zero because no PL family was detected by automated annotation.

Among GH families, GH13 was the most represented (13 genes, 24.5%). Members of GH13 encompass diverse catalytic subfamilies involved in α-glucan hydrolysis, such as α-amylases, α-N-acetylgalactosaminidases, and β-glycosidases, with double-displacement catalytic mechanisms (Lombard et al., 2014). Several predicted subfamilies (GH13_9, GH13_11, GH13_18, GH13_23, GH13_26, GH13_30, GH13_31) have been linked to hydrolysis of α-glucosidic bonds or α-1,3-L-(3,6-anhydro)-galactosidic linkages found in sulfated algal polysaccharides (Chernysheva et al., 2019). Additional GH families (GH1, GH3, GH5) were also identified, previously associated with the breakdown of laminarin oligomers into glucose in marine bacteria (Kalenborn et al., 2024).

GT families were highly abundant, with GT2 (28 genes, 40%) and GT4 (11 genes, 15.7%) dominating the repertoire (Fig. 4B). This distribution is consistent with marine microbial genomes, where GT2 and GT4 together typically account for more than half of annotated GTs (Chen et al., 2024). High GT abundance suggests a broad capacity for glycosidic bond formation and remodeling of cell surface or extracellular carbohydrates.

The genome also encoded auxiliary enzymes that may support polysaccharide utilization. AA3 oxidoreductases (five of eight AA genes) can act on glycosidic linkages and facilitate hydrolysis of complex substrates (Parab & Manohar, 2024). CBM families included CBM50 and CBM67, which may contribute to protein-carbohydrate interactions; CBM67 has been associated with the recognition of L-arabinose-containing structures. CE1 and CE4 families encode carbohydrate esterases that may contribute to the deacetylation of complex carbohydrates and increase substrate accessibility. A CE11-family protein annotated as a UDP-3-O-acyl-N-acetylglucosamine deacetylase, an enzyme involved in lipid A biosynthesis, was also detected.

No polysaccharide lyase (PL) family was detected by the automated dbCAN3 annotation pipeline. However, genome annotation identified one sulfatase gene belonging to family S1_7 in the SulfAtlas database, containing the conserved FGly activation motif. Members of this family are known to catalyze the hydrolysis of sulfate esters in various sulfated polysaccharides. Such enzymes may participate in the desulfation of sulfated oligosaccharides generated during polysaccharide degradation.

### Prediction of polysaccharide utilization loci (PUL)

#### Putative polysaccharide utilization cluster

Genome annotation of *Celeribacter* sp. ULVN23_4 revealed a candidate ulvan-associated gene cluster located on contig 3, representing a non-canonical, PUL-like genomic region. This locus encodes a set of enzymes previously implicated in the degradation of sulfated green algal polysaccharides, including a putative PL25-like protein, a glycoside hydrolase (GH105), and an S1_7-type sulfatase, which are further analyzed in the following section. The co-occurrence of these genes supports the interpretation that this region may function as a coordinated genomic module involved in the processing of ulvan-derived or related sulfated polysaccharides. However, the specific enzymatic activities and functional interactions of these components remain to be experimentally validated.

In addition to candidate ulvan-associated proteins, the locus contains genes encoding glycosyltransferases (GTs) and a complete ABC-type transporter, comprising a permease subunit and an ATP-binding protein, which are positioned adjacent to the candidate enzyme-coding genes. Notably, canonical SusC/D homologs typically found in Bacteroidetes PULs are absent, indicating that this locus represents a transporter-centered, non-canonical PUL-like system. Similar transporter-based architectures have been reported in Proteobacteria and are thought to function as functional analogs of the SusC/D-dependent paradigm, enabling the uptake of oligosaccharides generated extracellularly or within the periplasm (Grondin et al., 2017; Glowacki & Martens, 2021).

The genomic structure of this ulvan-related locus and its hypothetical model of the potential processing of an ulvan-derived substrate on a simulated structural segment of *Ulva lactuca* are illustrated in Figs. 5A and 5B, respectively, while detailed gene-level annotations are provided in Table 3. Taken together, these features support the identification of a putative PUL-like gene cluster potentially associated with ulvan utilization in *Celeribacter* sp. ULVN23_4. The identification of this ulvan-associated cluster provides the genomic basis for the detailed enzyme analyses presented in the following sections.

**Figure 5:**
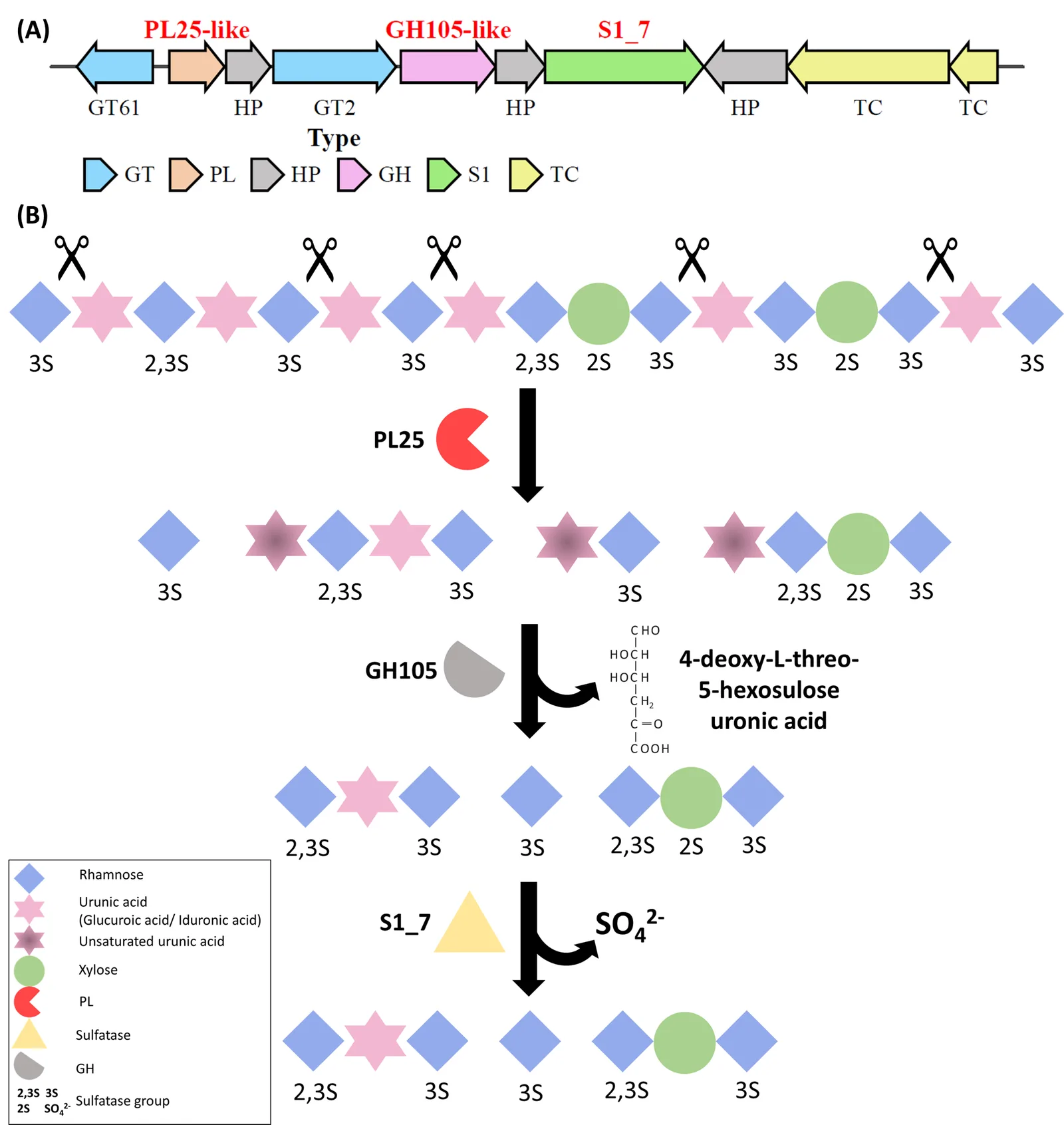
Organization and proposed functional model of a non-canonical ulvan-associated PUL-like locus in *Celeribacter* sp. ULVN23_4. (A) Gene organization of the locus, including genes encoding a putative PL25-like polysaccharide lyase, a putative GH105-like glycoside hydrolase, a putative S1_7-type sulfatase, putative glycosyltransferases (GTs), hypothetical proteins (HPs), and putative ABC-type transporter components (TC). Each arrow represents a gene and indicates its orientation. The corresponding locus IDs, strand orientations, and predicted functions are provided in Table 3, with genes listed in the same left-to-right order as in (A). Colors distinguish the predicted functional categories. (B) Proposed model of ulvan degradation involving the putative PL25, GH105, and S1_7 enzymes. The depicted reaction sequence is hypothetical. Numbers preceding “S” indicate the positions of sulfate substitutions on the sugar residues.

**Table 3:** Locus annotation of the ulvan-associated PUL-like gene cluster in *Celeribacter* sp. ULVN23_4. Gene annotations are based on genome annotation and functional prediction using CAZyme databases and sequence similarity searches. Locus IDs correspond to annotated coding sequences in the draft genome of strain ULVN23_4. Abbreviations: GT, glycosyltransferase; GH, glycoside hydrolase; PL, polysaccharide lyase; S1_7, sulfatase family; ABC, ATP-binding cassette transporter; HP, hypothetical protein.

**Locus ID**	**Strand**	**Feature**
LOCUS_20860	–	Glycosyltransferase family 61 protein
LOCUS_20870	+	Putative polysaccharide lyase family 25 protein
LOCUS_20880	+	Hypothetical protein
LOCUS_20890	+	Glycosyltransferase family 2 protein
LOCUS_20900	+	Putative glycoside hydrolase family 105 protein
LOCUS_20910	+	Hypothetical protein
LOCUS_20920	+	Sulfatase family S1_7 protein
LOCUS_20930	–	Hypothetical protein
LOCUS_20940	–	ABC transporter permease
LOCUS_20950	–	ABC transporter ATP-binding protein

To assess the genomic distribution and conservation of the putative ulvan-associated locus, a targeted comparative gene-neighborhood analysis was performed using 17 publicly available *Celeribacter* genomes representing the closest relatives of strain ULVN23_4 in the genome phylogeny. High-confidence homologs of all three anchor genes (LOCUS_20870, LOCUS_20900 and LOCUS_20920) were identified only in three strains, *C. persicus* DSM 100434, *C. ethanolicus* TSPH2, and *C. ethanolicus* NH195, showing 78.1–97.8% amino-acid identity with approximately complete query coverage. In these genomes, the three homologs were co-localized with adjacent transporter genes and displayed broadly conserved gene order and transcriptional orientation relative to strain ULVN23_4 (Fig. S4). In contrast, the remaining genomes lacked the complete co-localization of PL25-like, GH105-like, sulfatase, and adjacent transporter genes observed in ULVN23_4, indicating that this conserved putative PUL-like organization is restricted to a subset of closely related *Celeribacter* genomes. A summary of BLASTp results and genomic coordinates is provided in Table S4.

#### A putative PL25-like protein

No PL families were detected using the recommended dbCAN3 thresholds. However, exploratory analysis identified one candidate protein (LOCUS_20870) for further investigation. BLASTp analysis indicated remote similarity between LOCUS_20870 and experimentally characterized PL25 ulvan lyases, with approximately 25–38% identity over the aligned regions. Phylogenetic analysis further positioned LOCUS_20870 within a clade containing reference PL25 proteins (bootstrap = 85), clustering with reference PL25 enzymes that include experimentally characterized ulvan lyases from *Alteromonas* sp. KUL42, *Algibacter luteus*, and *Alteromonas* sp. TK-46(2) (Fig. 6A). Domain architecture analysis using Pfam and InterProScan did not detect a canonical PL25 domain, suggesting that this protein may represent a protein with remote sequence similarity to characterized polysaccharide lyases. Ulvan lyases are known to exhibit substantial sequence divergence, and several characterized enzymes share relatively low sequence identity despite catalyzing similar reactions. For example, the ulvan lyase from *Pseudoalteromonas* sp. PLSV (PsPL) shares only 43.44%, 38.47%, and 31.58% sequence identity with homologs from *Alteromonas* sp. LOR (AMA19991.1), *Catenovulum agarivorans* DS2 15349 (EWH08847.1), and *Glaciecola* sp. (BAY00693.1), respectively, and only 11.5% identity with the ulvan lyase from *Pseudoalteromonas* sp. PLSV2 (Qin et al., 2018). Pairwise sequence identity among the analyzed PL25 proteins is summarized in Table S1.

**Figure 6:**
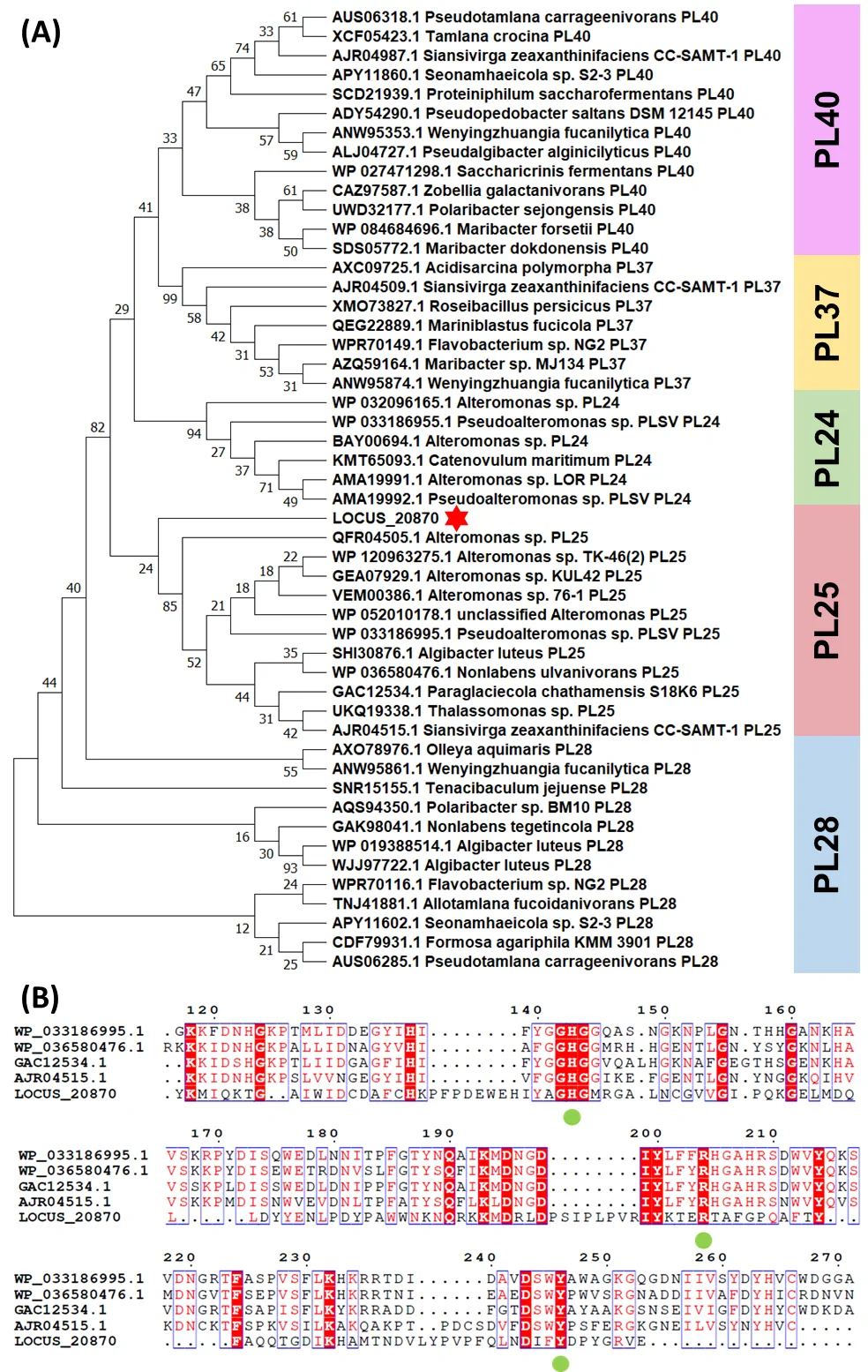
Sequence and phylogenetic analysis of a PL25-like protein (LOCUS_20870) from *Celeribacter* sp. ULVN23_4. (A) Maximum-likelihood phylogenetic tree of LOCUS_20870 and representative proteins from polysaccharide lyase families PL24, PL25, PL28, PL37, and PL40. Bootstrap values were calculated from 1000 replicates. (B) Multiple sequence alignment of LOCUS_20870 with characterized PL25 ulvan lyases. Positions corresponding to reported catalytic residues (His143, Arg204, and Tyr246) are indicated by symbols (green circles).

Multiple sequence alignment with representative PL25 enzymes identified His143, Arg204, and Tyr246 at positions corresponding to residues reported to contribute to catalysis in characterized PL25 ulvan lyases (Fig. 6B). In experimentally characterized PL25 enzymes, the corresponding residues are involved in active-site organization and the β-elimination mechanism (Ulaganathan et al., 2017; Gao et al., 2019). Although the overall amino acid sequence identity was low, the presence of these corresponding sequence features is compatible with, but does not establish, a related catalytic function in LOCUS_20870. Additional conserved sequence features identified by HMM-based analysis are summarized in Fig. S1.

Despite its supported placement in the selected phylogenetic analysis, the low sequence identity and the absence of a canonical PL25 domain preclude definitive assignment to the PL25 family. Therefore, LOCUS_20870 is conservatively described here as a putative PL25-like protein rather than as an assigned PL25 enzyme. Its sequence features and genomic context support a tentative evolutionary association with characterized PL25 proteins but do not establish catalytic activity or substrate specificity. Biochemical characterization will be required to determine whether LOCUS_20870 possesses polysaccharide-lyase activity and whether ulvan is a substrate.

#### A putative GH105-like protein

Adjacent to the gene encoding a putative PL25-like protein (LOCUS_20870), a gene encoding a putative GH105-like protein (LOCUS_20900) was identified. Members of this family are known as unsaturated uronyl hydrolases that cleave ΔUA residues generated by ulvan lyases.

Protein LOCUS_20900 shared 27–32% sequence identity with GH105-like enzymes from *Bacteroides thetaiotaomicron*, *Klebsiella pneumoniae*, and *Salmonella enterica*. Phylogenetic analysis placed LOCUS_20900 within the GH105 clade with strong bootstrap support (Fig. 7A).

**Figure 7:**
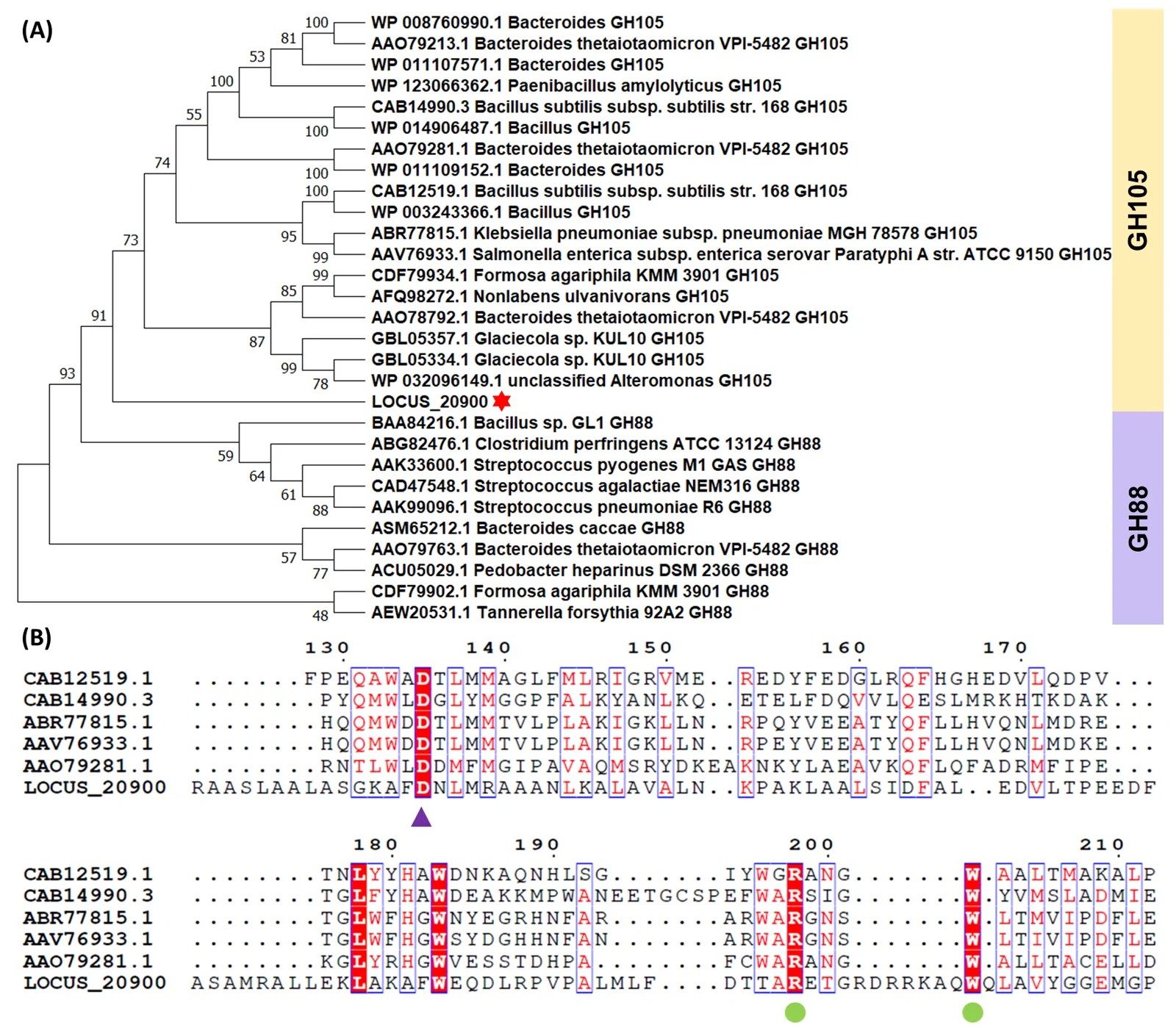
Sequence and phylogenetic analyses of a putative GH105 hydrolase (LOCUS_20900) from *Celeribacter* sp. ULVN23_4. (A) Maximum-likelihood phylogenetic tree of LOCUS_20900 and representative GH105 family enzymes. Bootstrap values were calculated from 1,000 replicates. (B) Multiple sequence alignment of LOCUS_20900 with characterized GH105 hydrolases. Positions corresponding to reported catalytic residues, including Asp135 (purple triangle) and the WxRxxxW motif involved in Δ UA (unsaturated uronic acid) recognition (green circles), are indicated by symbols.

Pairwise sequence identities among LOCUS_20900 and the representative GH105 proteins are summarized in Table S2. Multiple sequence alignment identified an Asp residue at the position corresponding to Asp135 reported as catalytic in characterized GH105 enzymes and the hallmark WxRxxxW motif required for ΔUA recognition (Fig. 7B). Pairwise sequence identity analysis and conserved motifs predicted from the Hidden Markov Model (HMM) profile are summarized in Fig. S2.

Although overall sequence identity to known GH105 enzymes was relatively low, the preservation of these catalytic motifs indicates possible conservation of selected sequence features. The genomic colocation of LOCUS_20900 with the PL25-like protein (LOCUS_20870) within the candidate ulvan-associated genomic region suggests a potential role in the downstream processing of unsaturated uronic acid products derived from ulvan.

#### S1_7 family sulfatase

Following the identification of a PUL-like cluster in ULVN23_4, the encoded sulfatase gene was examined for its potential role in ulvan degradation. Genome annotation revealed a single sulfatase gene (LOCUS_20920) assigned to subfamily S1_7, a group commonly acting on sulfated sugars such as those found in fucoidan and ulvan.

Multiple sequence alignment supported conservation of key catalytic residues, including the nucleophilic Cys60, Ca^2^^+^-coordinating Asp19, Asp20, Asp302, and Asn303 (Figs. 8A, 8C), as well as residues forming a putative sulfate - binding pocket (Arg64, His129, Lys111, His201, Lys314) (Fig. S3). These conserved sequence features, together with the phylogenetic placement of LOCUS_20920 within the clade containing reference S1_7 sulfatases (Fig. S5), support its predicted assignment to the S1_7 subfamily. The AlphaFold2 model of LOCUS_20920 showed high overall confidence, with a mean residue pLDDT of 92.5 (median 96.3), 78.1% of residues exhibiting pLDDT values above 90, and a predicted TM-score (pTM) of 0.94. The AlphaFold2-predicted structure of LOCUS_20920 contains α-helices and β-strands connected by loops, with the N- and C-termini indicated in Fig. 8B. Structural superposition with the experimentally determined S1_7 sulfatase from *Formosa agariphila* (PDB ID: 6HHM), identified as the closest structural template by SWISS-MODEL, yielded an RMSD of 1.072 Å over 1,250 aligned atoms, supporting substantial structural similarity. A close-up of the superimposed active-site regions is shown in Fig. 8C.

**Figure 8:**
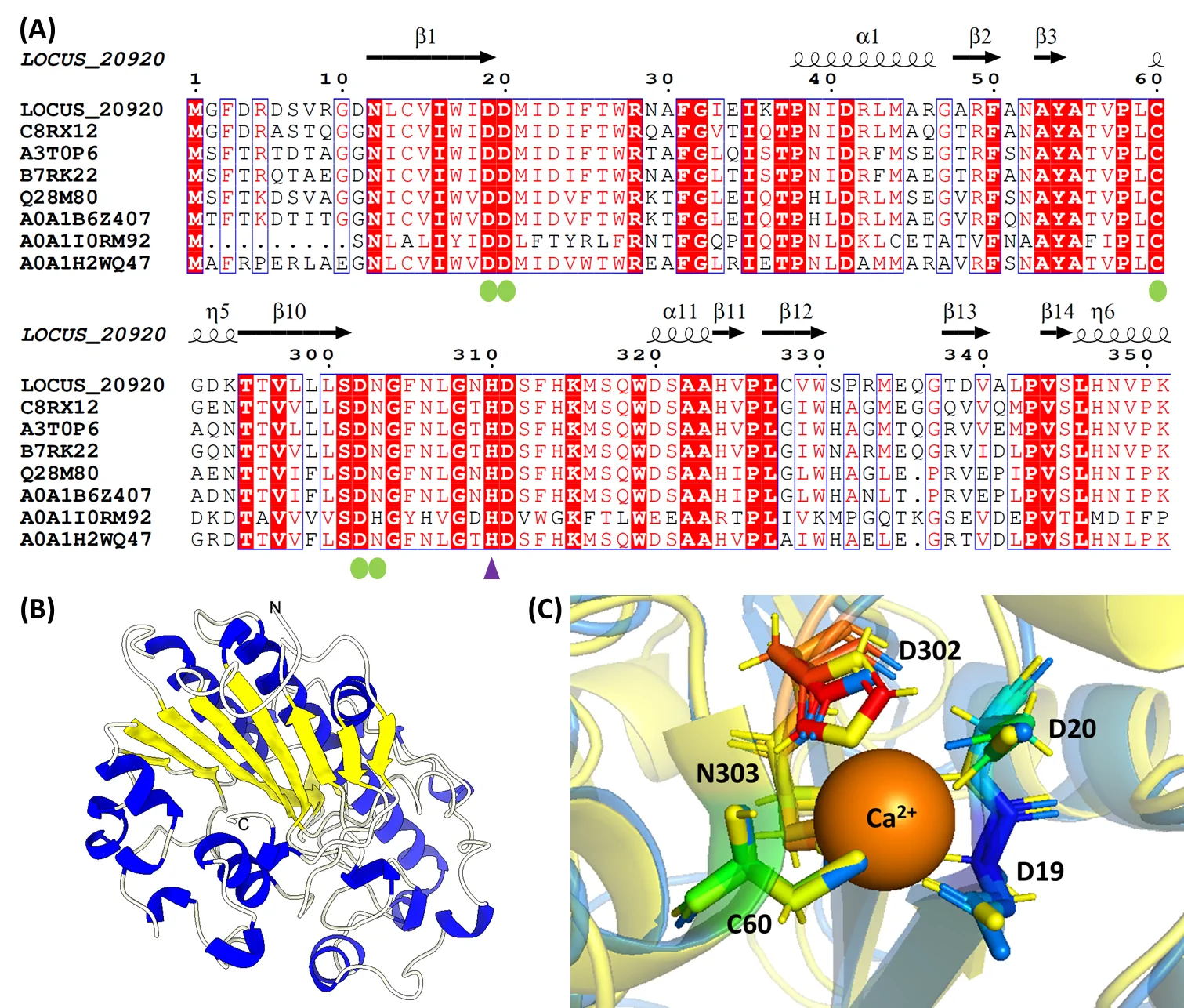
Sequence and structural analyses of the putative sulfatase LOCUS_20920 from *Celeribacter* sp. ULVN23_4. (A) Selected regions of a multiple sequence alignment of LOCUS_20920 with representative S1_7 sulfatases. Purple triangles and green circles mark positions corresponding to catalytic and Ca^2+^-coordinating residues, respectively, in the reference sulfatase. Secondary structure elements of LOCUS_20920 are indicated above the alignment. (B) Cartoon representation of the AlphaFold –predicted LOCUS_20920 model. Helices, β-strands, and connecting loops are shown in blue, yellow, and white, respectively. The N- and C-termini are labeled. (C) Close-up of the active-site regions following structural superposition of LOCUS_20920 (blue) and the experimentally determined S1_7 sulfatase from *Formosa agariphila* (PDB ID: 6HHM; yellow). Selected active-site residues are shown as sticks. The orange sphere represents the Ca^2+^ ion from 6HHM, serving as a reference for the putative calcium-binding site in LOCUS_20920. Residue labels follow LOCUS_20920 numbering.

Importantly, the extended pβH domain observed in LOCUS_20920 is consistent with adaptations in marine sulfatases that enable binding to bulky, flexible polysaccharide substrates. Such structural features suggest that LOCUS_20920 may contribute to the removal of sulfate groups from ulvan or related substrates, thereby exposing the glycosidic backbone for subsequent cleavage by polysaccharide lyases and hydrolases. Thus, the sulfatase LOCUS_20920 is consistent with those described for members of S1_7 enzymes and represents a candidate that may be involved in ulvan desulfation, based on conserved motifs and structural features in strain ULVN23_4.

Together, the presence of an S1_7 sulfatase (LOCUS_20920), a putative PL25-like protein (LOCUS_20870), and a putative GH105-like glycoside hydrolase (LOCUS_20900) within the same genomic region suggests that *Celeribacter* sp. ULVN23_4 contains a set of candidate genes that may function in a coordinated manner. This organization parallels the modular strategies described in marine Bacteroidetes and Alteromonadaceae, where sulfatases, lyases, and glycosidases act synergistically to deconstruct sulfated polysaccharides. In the proposed pathway, LOCUS_20920 may participate in ulvan desulfation, which could facilitate possible subsequent processing involving LOCUS_20870, while LOCUS_20900 may be involved in processing the resulting unsaturated uronic acids into utilizable monosaccharides. Such an arrangement may enable ULVN23_4 to utilize ulvan-derived substrates in marine environments, potentially contributing to carbon and sulfur cycling in coastal ecosystems. Although these predictions are consistent with the available sequence-based and genomic-context evidence, biochemical validation will be required to confirm substrate specificity and catalytic activity.

## Discussion

Although the gene cluster suggested in ULVN23_4 shares several features with polysaccharide utilization loci, its organization differs from the canonical PUL architecture described in Bacteroidetes. In particular, no SusC/D transporter pair or regulatory genes were detected within the locus, and SusC/D-like transporters are located elsewhere in the genome. Therefore, this region is more appropriately described as a PUL-like gene cluster rather than a classical PUL. Similar distributed architectures have been reported in several non-Bacteroidetes bacteria, where polysaccharide-degrading enzymes and transport systems are encoded in separate genomic regions.

Recent genomic and functional studies have suggested that several members of Proteobacteria possess polysaccharide utilization systems that are functionally analogous to classical Bacteroidetes polysaccharide utilization loci (PULs) but exhibit markedly distinct genomic architectures. In these systems, the canonical SusD component is frequently absent, and SusC-like functions are often replaced or complemented by TonB-dependent transporters (TBDTs), reflecting lineage-specific adaptations of outer membrane transport mechanisms in different bacterial groups (Grondin et al., 2017; Glowacki & Martens, 2021).

Representative examples include xylan-degrading gene clusters in *Caulobacter crescentus* (Alphaproteobacteria), where substrate uptake relies primarily on TBDTs acting in concert with polysaccharide-degrading enzymes (Bui et al., 2026), as well as alginate utilization loci in *Alteromonas macleodii* (Gammaproteobacteria), in which alginate depolymerization and uptake are mediated by TBDTs rather than classical SusC/D pairs (Neumann et al., 2015).

Notably, several polysaccharide utilization systems described to date display a pronounced spatial separation between depolymerizing enzymes and transport components while still enabling effective polysaccharide catabolism. In the reduced XyGUL system of *Cellvibrio japonicus*, extracellular enzymes involved in xyloglucan degradation are distributed across the genome and are not co-localized with transport genes, whereas oligosaccharide import depends on functionally coordinated TBDTs (DeBoy et al., 2008; McKee et al., 2021). Similarly, an atypical carrageenan-associated PUL described in the marine bacterium *Zobellia galactanivorans* lacks direct adjacency between core enzyme loci and SusC/D-like gene pairs; instead, distantly encoded transport components are transcriptionally induced during growth on carrageenan and function synergistically with depolymerizing enzymes (Ficko-Blean et al., 2017).

Consistent with these emerging models, the ulvan-associated gene cluster identified in *Celeribacter* sp. ULVN23_4 does not contain a contiguous SusC/D gene pair, a feature traditionally considered diagnostic of canonical Bacteroidetes PULs. Nevertheless, the genome of ULVN23_4 encodes putative SusD-like proteins (LOCUS_21370) and SusC-like TonB-dependent transporters located at genomic positions distant from the ulvan-associated locus (LOCUS_19460, LOCUS_23740). This dispersed genomic organization suggests a modular strategy in which ulvan depolymerization and oligosaccharide uptake are functionally linked but not necessarily encoded within a single contiguous locus. Such an arrangement does not necessarily imply reduced functional potential, but rather reflects architectural plasticity in polysaccharide utilization systems across bacterial lineages (Glowacki & Martens, 2021).

The ABC transporter encoded within the PUL-like locus may be involved in the transport of oligosaccharides across the inner membrane into the cytoplasm (Cuthbertson, Kos & Whitfield, 2010). Such a two-step transport strategy, involving outer membrane transporters and inner membrane ABC systems, has been described in Gram-negative bacteria such as *Xanthomonas campestris pv. campestris*, where sucrose transport involves comparable mechanisms. The absence of a local regulatory gene further indicates that this locus likely represents a non-canonical or distributed PUL-like module rather than a classical Bacteroidetes-type PUL (Blanvillain et al., 2007). Therefore, the putative ulvan-associated locus in ULVN23_4 likely reflects a distributed polysaccharide utilization system in which enzymatic depolymerization and carbohydrate transport are encoded in separate genomic regions. Comparative analysis indicated that the overall organization of the putative PUL-like locus is conserved only in three closely related *Celeribacter* strains. This observation suggests that the locus is restricted within a subset of the genus rather than being broadly distributed, consistent with its possible lineage-specific evolution.

Within this framework, the ulvan-associated gene cluster of *Celeribacter* sp. ULVN23_4 can be interpreted as a potential lineage-specific adaptation within Alphaproteobacteria, in which candidate ulvan-associated proteins are co-localized within the same genomic region, while substrate binding and uptake are potentially mediated by SusC/D-like and TonB-dependent transporters encoded elsewhere in the genome. From a comparative genomic perspective, the presence of a putative PL25-like protein within the ULVN23_4 locus is consistent with previously described ulvan utilization pathways in *Alteromonas*, *Nonlabens ulvanivorans*, *Pseudoalteromonas*, and *Thalassomonas* species, where PULs encoding a single PL25 have been reported (Ulaganathan et al., 2018; Wang et al., 2022). Notably, none of these previously characterized systems belong to Alphaproteobacteria, suggesting that putative ulvan-associated gene systems may also occur in members of this class, thereby extending the currently known phylogenetic distribution of ulvan utilization systems.

Nevertheless, the annotation of LOCUS_20870 as a putative PL25-like protein remains tentative. This prediction is supported by multiple computational observations, including remote sequence similarity to experimentally characterized ulvan lyases, phylogenetic placement within a PL25-related lineage, and partial conservation of characteristic motif residues. However, the relatively low sequence identity, the dependence of the phylogenetic placement on the selected reference dataset, and the absence of a canonical PL25 domain preclude definitive functional assignment. Notably, substantial sequence divergence has also been reported among experimentally characterized ulvan lyases (*e.g.*, *Alteromonas* sp. TK-45 (2) (WP_120963275.1) (Tang, Zhu & Yao, 2022), *Pseudoalteromonas* sp. PLSV (WP_033186995.1), *Alteromonas* sp.A321 (QFR04505.1) (Gao et al., 2019) suggesting that low sequence identity alone does not necessarily exclude functional relatedness. Therefore, the predicted function of LOCUS_20870 should be regarded as a hypothesis supported by multiple computational analyses rather than a confirmed enzymatic annotation, and additional structural and biochemical studies will be required to validate its enzymatic activity. Similarly, the annotation of LOCUS_20900 as a putative GH105-like protein remains tentative. Although its phylogenetic placement and sequence features are consistent with a possible relationship to GH105 enzymes, biochemical characterization is required to determine its catalytic activity and substrate specificity. Characterized GH105 enzymes act on unsaturated uronic acid-containing oligosaccharides derived from several polysaccharides; therefore, activity toward ulvan-derived products cannot be inferred solely from sequence annotation (Collén et al., 2014; Reisky et al., 2019).

Sulfatases represent another critical component of ulvan utilization, given that sulfate esters confer resistance to nonspecific hydrolysis and must be removed to enable downstream carbohydrate metabolism. The S1_7 sulfatase encoded within the ULVN23_4 locus belongs to a subfamily previously implicated in partial ulvan desulfation (Barbeyron et al., 2016; Stam et al., 2023). Although biochemical characterization of marine polysaccharide sulfatases remains comparatively limited relative to glycoside hydrolases and lyases, the presence of a conserved S1_7 sulfatase provides additional genome-based support for a candidate genomic region potentially associated with sulfated-polysaccharide processing in ULVN23_4 although substrate specificity toward ulvan remains to be experimentally validated.

From a genomic perspective, these findings suggest that members of the Alphaproteobacteria may harbor previously unrecognized genetic potential for ulvan utilization, thereby extending the current genomic evidence for putative ulvan-associated gene systems. The genome of *Celeribacter* sp. ULVN23_4 provides a useful reference for exploring alternative architectures of sulfated polysaccharide utilization systems and highlights the value of genome-based approaches for predicting metabolic potential in marine bacteria. Taken together, these findings provide a genomic framework for putative ulvan utilization in *Celeribacter* sp. ULVN23_4 rather than definitive evidence of enzymatic activity or pathway functionality.

## Conclusions

This study reports the isolation and genome analysis of *Celeribacter* sp. ULVN23_4, a putative novel Alphaproteobacterial genomic species obtained from the surface of *Ulva lactuca*. Comparative genomics supported its taxonomic distinctiveness, with ANI and dDDH values below species-level thresholds relative to reference *Celeribacter* species. Genome mining revealed a candidate ulvan-associated gene cluster composed of an S1_7 sulfatase, a putative PL25-like protein, and a putative GH105-like glycoside hydrolase co-localized with an ABC transporter. Plate-based assays provided phenotypic evidence of polysaccharide depolymerization and sulfatase-associated activity, which is consistent with, but does not confirm, the genomic predictions. Taken together, the genomic organization supports the hypothesis that this region may participate in the processing of ulvan-derived or related sulfated substrates. However, its function as a coordinated PUL-like system remains to be experimentally established. From a biotechnological perspective, the candidate proteins identified here, particularly the S1_7 sulfatase and the putative PL25-like protein, represent promising targets for future biochemical characterization. However, experimental validation will be required to confirm their substrate specificity and catalytic activity. Overall, our findings highlight *Celeribacter* sp. ULVN23_4 as a potential model for exploring ulvan-associated metabolism in Alphaproteobacteria. Future work, including recombinant expression, enzymatic assays, and transcriptomic analyses under ulvan-inducing conditions, will be essential to validate enzyme function and elucidate the regulatory mechanism.

## Supplemental Information

10.7717/peerj.21957/supp-1 Supplemental Information 1 HMM-based identification of conserved motifs in PL25 family proteins Hidden Markov Model (HMM) analysis of PL25 family proteins showing conserved amino acid motifs associated with catalytic activity and substrate binding. The HMM profile was generated using tools available at the DTU Health Tech bioinformatics services platform (https://services.healthtech.dtu.dk/).

10.7717/peerj.21957/supp-2 Supplemental Information 2 HMM-based identification of conserved motifs in GH105 family proteins Hidden Markov Model (HMM) analysis of GH105 family proteins showing conserved amino acid motifs associated with catalytic activity and substrate binding. The HMM profile was generated using tools available at the DTU Health Tech bioinformatics services platform (https://services.healthtech.dtu.dk/)

10.7717/peerj.21957/supp-3 Supplemental Information 3 Multiple sequence alignment of LOCUS_20920 with representative S1_7 sulfatases Multiple sequence alignment of LOCUS_20920 from Celeribacter sp. ULVN23_4 with representative S1_7 sulfatases. Conserved catalytic residues are indicated by purple triangles, and Ca^2+^-coordinating residues are indicated by green circles. Conserved amino acid regions support the assignment of LOCUS_20920 as a putative S1_7 family sulfatase potentially involved in the desulfation of ulvan or related sulfated polysaccharides.

10.7717/peerj.21957/supp-4 Supplemental Information 4 Comparative synteny of the putative ulvan-associated locus in *Celeribacter* sp. ULVN23_4 and closely related *Celeribacter* genomes Comparative gene-neighborhood analysis of the candidate ulvan-associated locus in *Celeribacter* sp. ULVN23_4 and three related genomes containing co-localized homologs of LOCUS_20870, LOCUS_20900, and LOCUS_20920: *C. ethanolicus* TSPH2 (GCA_002407265), *C. ethanolicus* NH195 (GCA_001550095), and *C. persicus* DSM 100434 (GCA_003050785). Arrows represent predicted protein-coding genes; arrow direction indicates transcriptional orientation, and arrow length is proportional to gene length. Genes are colored according to their predicted functions: GT, glycosyl transferase; PL25-like, putative PL25-like protein; HP, hypothetical protein; S1_7, S1_7 sulfatase; GH105-like, putative GH105-like protein; and TC, transporter component. Colored links connect homologous genes and indicate sequence similarity between genomic regions. The scale bar represents 2.5 kb.

10.7717/peerj.21957/supp-5 Supplemental Information 5 Phylogenetic analysis of the putative sulfatase LOCUS_20920 from *Celeribacter* sp. ULVN23_4 and reference sulfatases from ulvan-associated S1 subfamilies The analysis includes reference sequences from subfamilies S1_7, S1_8, and S1_25. LOCUS_20920 clusters with reference S1_7 sulfatases, supporting its predicted assignment to this subfamily.

10.7717/peerj.21957/supp-6 Supplemental Information 6 Pairwise sequence identity matrix of PL25 family proteins including LOCUS_20870 from *Celeribacter* sp. ULVN23_4 Pairwise amino acid sequence identity (%) among PL25 family proteins retrieved from the CAZy database and the candidate protein LOCUS_20870 from *Celeribacter* sp. ULVN23_4. Values represent percentage identity calculated from multiple sequence alignment.

10.7717/peerj.21957/supp-7 Supplemental Information 7 Pairwise amino acid sequence identity (%) among GH105 family proteins retrieved from the CAZy database and the candidate protein LOCUS_20900 from *Celeribacter* sp. ULVN23_4. Values represent percentage identity calculated from multiple sequences Pairwise amino acid sequence identity (%) among GH105 family proteins retrieved from the CAZy database and the candidate protein LOCUS_20900 from Celeribacter sp. ULVN23_4. Values represent percentage identity calculated from multiple sequence alignment.

10.7717/peerj.21957/supp-8 Supplemental Information 8 Computational workflow, bioinformatics software, and databases used for genome-based identification of a putative ulvan-related gene cluster in *Celeribacter* sp. ULVN23_4 Description of the computational workflow, bioinformatics software, databases, and analytical methods used for genome annotation, phylogenomic analysis, CAZyme prediction, sulfatase classification, PUL-like cluster identification, protein sequence analysis, structural prediction, and genome visualization in *Celeribacter* sp. ULVN23_4. No custom computer code or proprietary scripts were developed in this study.

10.7717/peerj.21957/supp-9 Supplemental Information 9 Distribution and genomic organization of homologs of the candidate ulvan-associated genes in representative *Celeribacter* genomes Homologs of LOCUS_20870, LOCUS_20900, and LOCUS_20920 were identified by BLASTp using an E-value threshold of 1 × 1^10^, minimum query coverage of 60%, and minimum amino-acid identity of 25%. For each retained hit, the protein accession, amino-acid identity, query coverage, E-value, bit score, genomic record, coordinates, and strand orientation are shown. “Component of conserved co-localized locus” indicates that homologs of all three candidate genes occur within the same genomic neighborhood together with adjacent transporter-associated genes. “Low-similarity hit” indicates that only a homolog of LOCUS_20870 was detected without co-localized LOCUS_20900 and LOCUS_20920 homologs. An em dash indicates that no hit met the specified retention criteria.

10.7717/peerj.21957/supp-10 Supplemental Information 10 Computational datasets and supporting files for phylogenetic, CAZyme, and comparative genomic analyses Computational datasets and supporting files used in this study. The archive contains the sequence alignments and phylogenetic analysis files, raw and summarized dbCAN3 CAZyme annotation outputs, BLASTp results and genomic-coordinate data used for gene-neighborhood analysis, comparative gene-cluster files, and a README document describing the contents, software, databases, and relevant analysis parameters.

## Additional Information and Declarations

### Field Study Permissions

The following information was supplied relating to field study approvals (i.e., approving body and any reference numbers): Field sampling was conducted under the institutional approval of the Vietnam Academy of Science and Technology (VAST) through research project VAST02.01/24-25, pursuant to Decision No. 1264/QÐ-VHL. The Institute of Oceanography, VAST, has additionally issued an official clarification confirming that no separate field-sampling permit from a governmental or local environmental management agency was required for this activity.

### Data Availability

The following information was supplied regarding data availability: The data is available in the Supplemental Files. The raw sequence data are available at NCBI BioProject: JBTVYO000000000.1.
