# Supplementary material for: Genome-based identification of a putative ulvan-related gene cluster in Celeribacter sp
Source: PeerJ. 2026 Nov 12;14:e21957. doi: 10.7717/peerj.21957 (PMC13770419; doi:10.7717/peerj.21957)
Supplement: Supplementary file 1 [file peerj-14-21957-s006.docx]

|  | **LOCUS_20870** | QXD23123.1 | AWB65560.1 | QGX63087.1 | XPP53918.1 | AJR04515.1 | QWG05426.1 | UKQ19338.1 | XER21115.1 | GAC12534.1 | GAC02962.1 | KHT64381.1 | WP_033186995.1 | WP_052010178.1 | VEM00386.1 | WP_120963275.1 | GEA07929.1 | TAP32765.1 | XLP09314.1 | XJJ52519.1 | QFR04505.1 | WAJ71959.1 | XIJ80461.1 |
| --- | --- | --- | --- | --- | --- | --- | --- | --- | --- | --- | --- | --- | --- | --- | --- | --- | --- | --- | --- | --- | --- | --- | --- |
| **LOCUS_20870** | 100 | 11.93 | 20.87 | 17.45 | 19.15 | 15.89 | 15.67 | 15.91 | 17.86 | 19.64 | 19.64 | 17.78 | 19.3 | 19.3 | 18.75 | 18.75 | 18.3 | 18.3 | 17.86 | 16.96 | 18.75 | 19.2 | 18.83 |
| QXD23123.1 | 11.93 | 100 | 18.77 | 17.95 | 16.92 | 17.88 | 19.55 | 20.22 | 21 | 23.42 | 23.16 | 22.28 | 20.74 | 19.95 | 20.86 | 21.35 | 21.66 | 21.66 | 22.99 | 21.28 | 21.72 | 22.87 | 21.89 |
| AWB65560.1 | 20.87 | 18.77 | 100 | 57.7 | 56.64 | 27.96 | 27.36 | 25.56 | 27.44 | 25.23 | 25.46 | 27.33 | 28 | 26.43 | 26.17 | 27.15 | 27.29 | 27.29 | 26.4 | 26.39 | 29.13 | 28.38 | 27.36 |
| QGX63087.1 | 17.45 | 17.95 | 57.7 | 100 | 71.72 | 29.63 | 29.37 | 27.74 | 31.11 | 29.2 | 29.43 | 30.34 | 30.24 | 30.28 | 28.6 | 29.73 | 29.07 | 29.07 | 28.63 | 28.3 | 31.88 | 29.22 | 31.89 |
| XPP53918.1 | 19.15 | 16.92 | 56.64 | 71.72 | 100 | 30.37 | 29.74 | 27.33 | 30.32 | 27.65 | 27.42 | 30.16 | 28.54 | 29.1 | 29.72 | 30 | 29.32 | 29.32 | 29.98 | 28.97 | 31.64 | 29.26 | 30.37 |
| AJR04515.1 | 15.89 | 17.88 | 27.96 | 29.63 | 30.37 | 100 | 62.98 | 40.67 | 43.99 | 41.72 | 41.27 | 42.28 | 41.2 | 42.21 | 42.51 | 42.08 | 41.83 | 41.83 | 42.28 | 41.56 | 45 | 43.9 | 43.21 |
| QWG05426.1 | 15.67 | 19.55 | 27.36 | 29.37 | 29.74 | 62.98 | 100 | 43.64 | 44.98 | 46.8 | 46.35 | 44.22 | 45.17 | 44.27 | 44.32 | 45.81 | 44.77 | 44.77 | 44.77 | 42.66 | 48.93 | 45.27 | 44.87 |
| UKQ19338.1 | 15.91 | 20.22 | 25.56 | 27.74 | 27.33 | 40.67 | 43.64 | 100 | 46.48 | 45.25 | 46.14 | 43.26 | 44.35 | 45.57 | 45.45 | 45.63 | 46.48 | 46.48 | 45.42 | 44.21 | 50.94 | 50.32 | 48.69 |
| XER21115.1 | 17.86 | 21 | 27.44 | 31.11 | 30.32 | 43.99 | 44.98 | 46.48 | 100 | 70 | 70.42 | 46.8 | 52.97 | 52.53 | 53.2 | 53.72 | 52.1 | 52.1 | 53.64 | 53.73 | 57.01 | 53.76 | 55.38 |
| GAC12534.1 | 19.64 | 23.42 | 25.23 | 29.2 | 27.65 | 41.72 | 46.8 | 45.25 | 70 | 100 | 97.51 | 48.79 | 52.64 | 51.98 | 51.11 | 51.92 | 50.88 | 50.88 | 52.43 | 51.21 | 54.21 | 53.32 | 55.92 |
| GAC02962.1 | 19.64 | 23.16 | 25.46 | 29.43 | 27.42 | 41.27 | 46.35 | 46.14 | 70.42 | 97.51 | 100 | 48.34 | 52.64 | 51.98 | 51.11 | 51.92 | 50.88 | 50.88 | 52.43 | 51.43 | 54.91 | 53.76 | 56.36 |
| KHT64381.1 | 17.78 | 22.28 | 27.33 | 30.34 | 30.16 | 42.28 | 44.22 | 43.26 | 46.8 | 48.79 | 48.34 | 100 | 50.98 | 50.98 | 50.11 | 52.2 | 50.33 | 50.33 | 49.89 | 50.22 | 56.09 | 55.27 | 49.78 |
| WP_033186995.1 | 19.3 | 20.74 | 28 | 30.24 | 28.54 | 41.2 | 45.17 | 44.35 | 52.97 | 52.64 | 52.64 | 50.98 | 100 | 73.6 | 72.97 | 75.86 | 72.56 | 72.56 | 69.85 | 60.17 | 63.74 | 62.08 | 57.3 |
| WP_052010178.1 | 19.3 | 19.95 | 26.43 | 30.28 | 29.1 | 42.21 | 44.27 | 45.57 | 52.53 | 51.98 | 51.98 | 50.98 | 73.6 | 100 | 88.05 | 87.34 | 84.49 | 84.49 | 81.34 | 60.59 | 61.43 | 60.86 | 54.47 |
| VEM00386.1 | 18.75 | 20.86 | 26.17 | 28.6 | 29.72 | 42.51 | 44.32 | 45.45 | 53.2 | 51.11 | 51.11 | 50.11 | 72.97 | 88.05 | 100 | 89.06 | 88.66 | 88.66 | 85.57 | 59.34 | 62.27 | 59.79 | 55.36 |
| WP_120963275.1 | 18.75 | 21.35 | 27.15 | 29.73 | 30 | 42.08 | 45.81 | 45.63 | 53.72 | 51.92 | 51.92 | 52.2 | 75.86 | 87.34 | 89.06 | 100 | 89.91 | 89.91 | 86.05 | 61.42 | 62.96 | 61.3 | 57.46 |
| GEA07929.1 | 18.3 | 21.66 | 27.29 | 29.07 | 29.32 | 41.83 | 44.77 | 46.48 | 52.1 | 50.88 | 50.88 | 50.33 | 72.56 | 84.49 | 88.66 | 89.91 | 100 | 100 | 91.13 | 60.46 | 61.81 | 59.87 | 55.8 |
| TAP32765.1 | 18.3 | 21.66 | 27.29 | 29.07 | 29.32 | 41.83 | 44.77 | 46.48 | 52.1 | 50.88 | 50.88 | 50.33 | 72.56 | 84.49 | 88.66 | 89.91 | 100 | 100 | 91.13 | 60.46 | 61.81 | 59.87 | 55.8 |
| XLP09314.1 | 17.86 | 22.99 | 26.4 | 28.63 | 29.98 | 42.28 | 44.77 | 45.42 | 53.64 | 52.43 | 52.43 | 49.89 | 69.85 | 81.34 | 85.57 | 86.05 | 91.13 | 91.13 | 100 | 59.01 | 60.42 | 58.17 | 55.58 |
| XJJ52519.1 | 16.96 | 21.28 | 26.39 | 28.3 | 28.97 | 41.56 | 42.66 | 44.21 | 53.73 | 51.21 | 51.43 | 50.22 | 60.17 | 60.59 | 59.34 | 61.42 | 60.46 | 60.46 | 59.01 | 100 | 64.29 | 59.04 | 55.87 |
| QFR04505.1 | 18.75 | 21.72 | 29.13 | 31.88 | 31.64 | 45 | 48.93 | 50.94 | 57.01 | 54.21 | 54.91 | 56.09 | 63.74 | 61.43 | 62.27 | 62.96 | 61.81 | 61.81 | 60.42 | 64.29 | 100 | 75.92 | 62.47 |
| WAJ71959.1 | 19.2 | 22.87 | 28.38 | 29.22 | 29.26 | 43.9 | 45.27 | 50.32 | 53.76 | 53.32 | 53.76 | 55.27 | 62.08 | 60.86 | 59.79 | 61.3 | 59.87 | 59.87 | 58.17 | 59.04 | 75.92 | 100 | 58.9 |
| XIJ80461.1 | 18.83 | 21.89 | 27.36 | 31.89 | 30.37 | 43.21 | 44.87 | 48.69 | 55.38 | 55.92 | 56.36 | 49.78 | 57.3 | 54.47 | 55.36 | 57.46 | 55.8 | 55.8 | 55.58 | 55.87 | 62.47 | 58.9 | 100 |
