# Supplementary material for: Genome-based identification of a putative ulvan-related gene cluster in Celeribacter sp
Source: PeerJ. 2026 Nov 12;14:e21957. doi: 10.7717/peerj.21957 (PMC13770419; doi:10.7717/peerj.21957)
Supplement: Supplementary file 2 [file peerj-14-21957-s007.docx]

|  | **LOCUS_20900** | AAO78792.1 | CDF79934.1 | AFQ98272.1 | GBL05357.1 | GBL05334.1 | WP_032096149.1 | CAB12519.1 | WP_003243366.1 | ABR77815.1 | AAV76933.1 | AAO79281.1 | WP_011109152.1 | CAB14990.3 | WP_014906487.1 | WP_123066362.1 | WP_008760990.1 | AAO79213.1 | WP_011107571.1 |
| --- | --- | --- | --- | --- | --- | --- | --- | --- | --- | --- | --- | --- | --- | --- | --- | --- | --- | --- | --- |
| **LOCUS_20900** | 100 | 16.18 | 13.97 | 15.69 | 14.09 | 17.63 | 16.61 | 14.06 | 14.06 | 16.15 | 15.86 | 17.19 | 17.19 | 14.23 | 14.23 | 17.56 | 16.1 | 16.1 | 13.95 |
| AAO78792.1 | 16.18 | 100 | 32.76 | 32.47 | 28.81 | 30.49 | 28.34 | 17.46 | 17.46 | 19.82 | 19.57 | 19.6 | 19.6 | 20.78 | 20.78 | 19.06 | 16.57 | 16.57 | 20.06 |
| CDF79934.1 | 13.97 | 32.76 | 100 | 72.27 | 35.2 | 33.7 | 33.97 | 20.45 | 20.45 | 16.72 | 17.39 | 21.16 | 21.16 | 22.15 | 22.15 | 19.18 | 18.6 | 18.6 | 19.59 |
| AFQ98272.1 | 15.69 | 32.47 | 72.27 | 100 | 34.36 | 35.79 | 36.14 | 20.92 | 20.92 | 18.07 | 18.75 | 22.9 | 22.9 | 21.85 | 21.85 | 18.93 | 17.44 | 17.44 | 20.76 |
| GBL05357.1 | 14.09 | 28.81 | 35.2 | 34.36 | 100 | 59.29 | 55.08 | 18.93 | 18.93 | 18.51 | 18.56 | 20.73 | 20.73 | 22.82 | 22.82 | 21.67 | 16.38 | 16.38 | 19.89 |
| GBL05334.1 | 17.63 | 30.49 | 33.7 | 35.79 | 59.29 | 100 | 62.35 | 23.34 | 23.34 | 20.23 | 20 | 23.59 | 23.59 | 20.24 | 20.24 | 22.8 | 15.41 | 15.41 | 18.87 |
| WP_032096149.1 | 16.61 | 28.34 | 33.97 | 36.14 | 55.08 | 62.35 | 100 | 22.33 | 22.33 | 19.3 | 19.65 | 22.25 | 22.25 | 19.29 | 19.29 | 19.45 | 17.17 | 17.17 | 18.38 |
| CAB12519.1 | 14.06 | 17.46 | 20.45 | 20.92 | 18.93 | 23.34 | 22.33 | 100 | 100 | 29.67 | 28.49 | 21.39 | 21.39 | 24.22 | 24.22 | 23.9 | 22.36 | 22.36 | 22.6 |
| WP_003243366.1 | 14.06 | 17.46 | 20.45 | 20.92 | 18.93 | 23.34 | 22.33 | 100 | 100 | 29.67 | 28.49 | 21.39 | 21.39 | 24.22 | 24.22 | 23.9 | 22.36 | 22.36 | 22.6 |
| ABR77815.1 | 16.15 | 19.82 | 16.72 | 18.07 | 18.51 | 20.23 | 19.3 | 29.67 | 29.67 | 100 | 87.04 | 21.8 | 21.8 | 22.92 | 22.92 | 26.49 | 23.85 | 23.85 | 25 |
| AAV76933.1 | 15.86 | 19.57 | 17.39 | 18.75 | 18.56 | 20 | 19.65 | 28.49 | 28.49 | 87.04 | 100 | 22.95 | 22.95 | 23.58 | 23.58 | 25.89 | 23.63 | 23.63 | 26.51 |
| AAO79281.1 | 17.19 | 19.6 | 21.16 | 22.9 | 20.73 | 23.59 | 22.25 | 21.39 | 21.39 | 21.8 | 22.95 | 100 | 100 | 23.21 | 23.21 | 24.71 | 23.92 | 23.92 | 24.8 |
| WP_011109152.1 | 17.19 | 19.6 | 21.16 | 22.9 | 20.73 | 23.59 | 22.25 | 21.39 | 21.39 | 21.8 | 22.95 | 100 | 100 | 23.21 | 23.21 | 24.71 | 23.92 | 23.92 | 24.8 |
| CAB14990.3 | 14.23 | 20.78 | 22.15 | 21.85 | 22.82 | 20.24 | 19.29 | 24.22 | 24.22 | 22.92 | 23.58 | 23.21 | 23.21 | 100 | 100 | 38.14 | 35.23 | 35.23 | 40.27 |
| WP_014906487.1 | 14.23 | 20.78 | 22.15 | 21.85 | 22.82 | 20.24 | 19.29 | 24.22 | 24.22 | 22.92 | 23.58 | 23.21 | 23.21 | 100 | 100 | 38.14 | 35.23 | 35.23 | 40.27 |
| WP_123066362.1 | 17.56 | 19.06 | 19.18 | 18.93 | 21.67 | 22.8 | 19.45 | 23.9 | 23.9 | 26.49 | 25.89 | 24.71 | 24.71 | 38.14 | 38.14 | 100 | 41.14 | 41.14 | 43.32 |
| WP_008760990.1 | 16.1 | 16.57 | 18.6 | 17.44 | 16.38 | 15.41 | 17.17 | 22.36 | 22.36 | 23.85 | 23.63 | 23.92 | 23.92 | 35.23 | 35.23 | 41.14 | 100 | 100 | 46.1 |
| AAO79213.1 | 16.1 | 16.57 | 18.6 | 17.44 | 16.38 | 15.41 | 17.17 | 22.36 | 22.36 | 23.85 | 23.63 | 23.92 | 23.92 | 35.23 | 35.23 | 41.14 | 100 | 100 | 46.1 |
| WP_011107571.1 | 13.95 | 20.06 | 19.59 | 20.76 | 19.89 | 18.87 | 18.38 | 22.6 | 22.6 | 25 | 26.51 | 24.8 | 24.8 | 40.27 | 40.27 | 43.32 | 46.1 | 46.1 | 100 |
