# Supplementary material for: Genome-based identification of a putative ulvan-related gene cluster in Celeribacter sp
Source: PeerJ. 2026 Nov 12;14:e21957. doi: 10.7717/peerj.21957 (PMC13770419; doi:10.7717/peerj.21957)
Supplement: Supplementary file 3 [file peerj-14-21957-s008.docx]

| **Analysis** | **Software / Database** | **Version / Platform** | **Method / command** |
| --- | --- | --- | --- |
| Genome annotation | RAST, DFAST, BV-BRC | Web server | Genome annotation performed using public annotation servers |
| Functional annotation | eggNOG-mapper | v6.0 | Public software |
| 16S rRNA phylogeny | MEGA | v11 | Neighbor-Joining tree with 1000 bootstrap replicates |
| Core gene phylogenomics | UBCG, MAFFT, RAxML | Public pipeline | Public software |
| ANI analysis | OrthoANI | Software | Public software |
| dDDH analysis | GGDC | v3.0 web server | Formula 2 used for species delineation |
| CAZyme prediction | dbCAN3 | Web server | HMMER and DIAMOND searches against CAZy/dbCAN |
| Sulfatase classification | SulfAtlas | Web database | HMMER search against SulfAtlas HMM profiles |
| PUL prediction | dbCAN-PUL | Web server | Identification based on CAZyme and transporter co-localization |
| Phylogenetic tree visualization | iTOL | Web server | Public software |
| Gene cluster visualization | Chiplot | Web server | Public software |
| Multiple sequence alignment | Clustal Omega, MAFFT | Web server | Public software |
| Protein phylogeny | MEGA | v11 | Maximum likelihood tree with 1000 bootstraps |
| Structure prediction | AlphaFold2, SWISS-MODEL | Web platform | Protein structural prediction |
| Structure visualization | PyMOL | v2.5 | Structural visualization and comparison |
| Functional domain analysis | Pfam, InterProScan | Web server | Public software |
| Genome visualization | Proksee | Web server | Circular genome map generation |

All analyses were conducted using publicly available software, web servers, and databases with default or recommended parameters unless otherwise stated in the main text
