# Supplementary material for: Genome-based identification of a putative ulvan-related gene cluster in Celeribacter sp
Source: PeerJ. 2026 Nov 12;14:e21957. doi: 10.7717/peerj.21957 (PMC13770419; doi:10.7717/peerj.21957)
Supplement: Supplementary file 4 [file peerj-14-21957-s009.docx]

**Table S4**. Distribution and genomic organization of the putative ulvan-associated locus in representative *Celeribacter* genomes.

| **Genome accession** | **Organism /** | **Query** | **Protein ID** | **Identity (%)** | **Query coverage per Subject (%)** | **E-value** | **Bitscore** | **Record / contig** | **Start** | **End** | **Strand** | **Interpretation** |
| --- | --- | --- | --- | --- | --- | --- | --- | --- | --- | --- | --- | --- |
| GCA_000819565 | *Celeribacter indicus* P73 | LOCUS_20870 | WP_052453031.1 | 30.258 | 100 | 1.56E-35 | 127.0 | NZ_CP004393.1 | 793886 | 794905 | + | Low-similarity hit |
| GCA_000819565 | *Celeribacter indicus* P73 | LOCUS_20900 | — |  |  | — |  | — |  |  | — | No retained hit |
| GCA_000819565 | Celeribacter indicus P73 | LOCUS_20920 | — |  |  | — |  | — |  |  | — | No retained hit |
| GCA_001308265 | *Celeribacter marinus* IMCC12053 | LOCUS_20870 | WP_062216513.1 | 30.882 | 100 | 8.19E-39 | 135.0 | NZ_CP012023.1 | 1141079 | 1142092 | - | Low-similarity hit |
| GCA_001308265 | *Celeribacter marinus* IMCC12053 | LOCUS_20900 | — |  |  | — |  | — |  |  | — | No retained hit |
| GCA_001308265 | *Celeribacter marinus* IMCC12053 | LOCUS_20920 | — |  |  | — |  | — |  |  | — | No retained hit |
| GCA_001550085 | *Celeribacter halophilus* ZXM137 | LOCUS_20870 | WP_066608293.1 | 31.387 | 100 | 1.70E-37 | 132.0 | NZ_LRUD01000068.1 | 63899 | 64903 | + | Low-similarity hit |
| GCA_001550085 | *Celeribacter halophilus* ZXM137 | LOCUS_20900 | — |  |  | — |  | — |  |  | — | No retained hit |
| GCA_001550085 | *Celeribacter halophilus* ZXM137 | LOCUS_20920 | — |  |  | — |  | — |  |  | — | No retained hit |
| GCA_001550095 | *Celeribacter ethanolicus* NH195 | LOCUS_20870 | WP_081423937.1 | 97.398 | 100 | 0 | 558.0 | NZ_LRUC01000038.1 | 22078 | 22887 | - | Component of conserved co-localized locus |
| GCA_001550095 | *Celeribacter ethanolicus* NH195 | LOCUS_20900 | WP_066708112.1 | 78.139 | 100 | 0 | 710.0 | NZ_LRUC01000038.1 | 18810 | 20192 | - | Component of conserved co-localized locus |
| GCA_001550095 | *Celeribacter ethanolicus* NH195 | LOCUS_20920 | WP_066708106.1 | 92.428 | 98.966 | 0 | 1444.0 | NZ_LRUC01000038.1 | 15765 | 18089 | - | Component of conserved co-localized locus |
| GCA_002341365 | *Celeribacter baekdonensis* UBA2508 | LOCUS_20870 | WP_152002031.1 | 31.716 | 99.628 | 2.08E-35 | 130.0 | NZ_DDNY01000005.1 | 31695 | 33563 | - | Low-similarity hit |
| GCA_002341365 | *Celeribacter baekdonensis* UBA2508 | LOCUS_20900 | — |  |  | — |  | — |  |  | — | No retained hit |
| GCA_002341365 | *Celeribacter baekdonensis* UBA2508 | LOCUS_20920 | — |  |  | — |  | — |  |  | — | No retained hit |
| GCA_002407265 | *Celeribacter ethanolicus* TSPH2 | LOCUS_20870 | WP_096805684.1 | 97.026 | 100 | 0 | 557.0 | NZ_CP022196.1 | 1831046 | 1831855 | - | Component of conserved co-localized locus |
| GCA_002407265 | *Celeribacter ethanolicus* TSPH2 | LOCUS_20900 | WP_096805683.1 | 78.355 | 100 | 0 | 714.0 | NZ_CP022196.1 | 1827778 | 1829160 | - | Component of conserved co-localized locus |
| GCA_002407265 | *Celeribacter ethanolicus* TSPH2 | LOCUS_20920 | WP_096805681.1 | 92.167 | 98.966 | 0 | 1438.0 | NZ_CP022196.1 | 1824733 | 1827057 | - | Component of conserved co-localized locus |
| GCA_003050785 | *Celeribacter persicus* DSM 100434 | LOCUS_20870 | WP_107815740.1 | 97.770 | 100 | 0 | 558.0 | NZ_QAOH01000004.1 | 44754 | 45563 | - | Component of conserved co-localized locus |
| GCA_003050785 | *Celeribacter persicus* DSM 100434 | LOCUS_20900 | WP_107815739.1 | 78.280 | 100 | 0 | 694.0 | NZ_QAOH01000004.1 | 41473 | 42870 | - | Component of conserved co-localized locus |
| GCA_003050785 | *Celeribacter persicus* DSM 100434 | LOCUS_20920 | WP_107815737.1 | 90.260 | 99.483 | 0 | 1429.0 | NZ_QAOH01000004.1 | 38416 | 40752 | - | Component of conserved co-localized locus |
| GCA_003254175 | *Celeribacter halophilus* DSM 26270 | LOCUS_20870 | WP_066608293.1 | 31.387 | 100 | 1.69E-37 | 132.0 | NZ_QKZJ01000013.1 | 80322 | 81326 | + | Low-similarity hit |
| GCA_003254175 | *Celeribacter halophilus* DSM 26270 | LOCUS_20900 | — |  |  | — |  | — |  |  | — | No retained hit |
| GCA_003254175 | *Celeribacter halophilus* DSM 26270 | LOCUS_20920 | — |  |  | — |  | — |  |  | — | No retained hit |
| GCA_018860305 | *Celeribacter halophilus* G3M19 | LOCUS_20870 | WP_216043817.1 | 31.387 | 100 | 2.75E-39 | 136.0 | NZ_JAHKPE010000018.1 | 39549 | 40553 | - | Low-similarity hit |
| GCA_018860305 | *Celeribacter halophilus* G3M19 | LOCUS_20900 | — |  |  | — |  | — |  |  | — | No retained hit |
| GCA_018860305 | *Celeribacter halophilus* G3M19 | LOCUS_20920 | — |  |  | — |  | — |  |  | — | No retained hit |
| GCA_020165855 | *Celeribacter litoreus* ASW11-22 | LOCUS_20870 | WP_224503701.1 | 33.043 | 85.502 | 8.83E-35 | 125.0 | NZ_JAIUFY010000013.1 | 311123 | 312205 | + | Low-similarity hit |
| GCA_020165855 | *Celeribacter litoreus* ASW11-22 | LOCUS_20900 | — |  |  | — |  | — |  |  | — | No retained hit |
| GCA_020165855 | *Celeribacter litoreus* ASW11-22 | LOCUS_20920 | — |  |  | — |  | — |  |  | — | No retained hit |
| GCA_020524585 | *Celeribacter naphthalenivorans* EaN35-2 | LOCUS_20870 | WP_226552312.1 | 32.090 | 99.628 | 5.30E-40 | 138.0 | NZ_JAIVLK010000004.1 | 14400 | 15404 | + | Low-similarity hit |
| GCA_020524585 | *Celeribacter naphthalenivorans* EaN35-2 | LOCUS_20900 | — |  |  | — |  | — |  |  | — | No retained hit |
| GCA_020524585 | *Celeribacter naphthalenivorans* EaN35-2 | LOCUS_20920 | — |  |  | — |  | — |  |  | — | No retained hit |
| GCA_030547445 | *Celeribacter halophilus* F3R18 | LOCUS_20870 | WP_216043817.1 | 31.387 | 100 | 2.75E-39 | 136.0 | NZ_JAUOPY010000013.1 | 39420 | 40424 | - | Low-similarity hit |
| GCA_030547445 | *Celeribacter halophilus* F3R18 | LOCUS_20900 | — |  |  | — |  | — |  |  | — | No retained hit |
| GCA_030547445 | *Celeribacter halophilus* F3R18 | LOCUS_20920 | — |  |  | — |  | — |  |  | — | No retained hit |
| GCA_030548225 | *Celeribacter halophilus* B3M18 | LOCUS_20870 | WP_303484627.1 | 31.387 | 100 | 5.62E-39 | 135.0 | NZ_JAUOSA010000015.1 | 39331 | 40335 | + | Low-similarity hit |
| GCA_030548225 | *Celeribacter halophilus* B3M18 | LOCUS_20900 | — |  |  | — |  | — |  |  | — | No retained hit |
| GCA_030548225 | *Celeribacter halophilus* B3M18 | LOCUS_20920 | — |  |  | — |  | — |  |  | — | No retained hit |
| GCA_036697285 | *Celeribacter halophilus* 14N.309.X.WAT.G.D12 | LOCUS_20870 | WP_332937969.1 | 30.657 | 100 | 6.79E-37 | 130.0 | NZ_JBAHYZ010000030.1 | 14173 | 15177 | - | Low-similarity hit |
| GCA_036697285 | *Celeribacter halophilus* 14N.309.X.WAT.G.D12 | LOCUS_20900 | — |  |  | — |  | — |  |  | — | No retained hit |
| GCA_036697285 | *Celeribacter halophilus* 14N.309.X.WAT.G.D12 | LOCUS_20920 | — |  |  | — |  | — |  |  | — | No retained hit |
| GCA_039539485 | *Celeribacter arenosi* JCM 17190 | LOCUS_20870 | WP_344846204.1 | 31.579 | 98.885 | 2.27E-40 | 139.0 | NZ_BAABDF010000007.1 | 124848 | 125882 | + | Low-similarity hit |
| GCA_039539485 | *Celeribacter arenosi* JCM 17190 | LOCUS_20900 | — |  |  | — |  | — |  |  | — | No retained hit |
| GCA_039539485 | *Celeribacter arenosi* JCM 17190 | LOCUS_20920 | — |  |  | — |  | — |  |  | — | No retained hit |
| GCA_900113955 | *Celeribacter neptunius* DSM 26471 | LOCUS_20870 | WP_090060260.1 | 35.169 | 87.732 | 1.93E-50 | 164.0 | NZ_FORH01000003.1 | 89157 | 89957 | + | Low-similarity hit |
| GCA_900113955 | *Celeribacter neptunius* DSM 26471 | LOCUS_20900 | — |  |  | — |  | — |  |  | — | No retained hit |
| GCA_900113955 | *Celeribacter neptunius* DSM 26471 | LOCUS_20920 | — |  |  | — |  | — |  |  | — | No retained hit |
| GCA_900114135 | *Celeribacter halophilus* CGMCC 1.8891 | LOCUS_20870 | WP_066608293.1 | 31.387 | 100 | 1.69E-37 | 132.0 | NZ_FORY01000020.1 | 23192 | 24196 | + | Low-similarity hit |
| GCA_900114135 | *Celeribacter halophilus* CGMCC 1.8891 | LOCUS_20900 | — |  |  | — |  | — |  |  | — | No retained hit |
| GCA_900114135 | *Celeribacter halophilus* CGMCC 1.8891 | LOCUS_20920 | — |  |  | — |  | — |  |  | — | No retained hit |
